# Semi-supervised Omics Factor Analysis (SOFA) disentangles known and latent sources of variation in multi-omic data

**DOI:** 10.1038/s41467-026-74694-6

**Published:** 2026-08-20

**Authors:** Tümay Capraz, Harald Vöhringer, Klaus Sebastian Augusto Kruger Serrano, Ricardo Omar Ramirez Flores, Julio Saez-Rodriguez, Wolfgang Huber

**Affiliations:** 1 Collaboration for joint PhD degree between EMBL and Heidelberg University, Faculty of Biosciences, Heidelberg, Germany; 2 Genome Biology Unit, EMBL, Heidelberg, Germany; 3 Molecular Medicine Partnership Unit (MMPU), Heidelberg, Germany; 4 Department of Medicine V, Hematology, Oncology and Rheumatology, University Hospital Heidelberg, Heidelberg, Germany; 5 Department of Hematology and Oncology, University Hospital Düsseldorf, Düsseldorf, Germany; 6 Heidelberg University, Faculty of Medicine, and Heidelberg University Hospital, Institute for Computational Biomedicine, Heidelberg, Germany; 7 European Molecular Biology Laboratory, European Bioinformatics Institute (EMBL-EBI), Hinxton, UK

**Keywords:** Data integration, Software, Statistical methods

## Abstract

A fundamental design pattern in biomolecular studies is to assay the same set of samples (organisms, tissue biopsies, or individual cells) by multiple different ‘omics assays. Group Factor Analysis (GFA) and its adaptation to high-dimensional settings, Multi-Omics Factor Analysis (MOFA), are widely used as a first-line approach to analyze such data and are effective in detecting patterns of correlation, organize them into so-called latent factors, and identify common and assay-specific factors. However, in many applications, a subset of the found factors just rediscovers already known covariates (e.g., disease subtypes, environmental covariates) while others may represent genuine novelty.

Here, we present Semi-supervised Omics Factor Analysis (SOFA), a method that incorporates known covariates into the model upfront and focuses the factor discovery on novel sources of variation. We show SOFA’s effectiveness for discovering novel patterns by applying it to cancer, brain development and heart failure multi-omic data sets.

## Abstract (web summary)

Capraz et al. introduce Semi-supervised Omics Factor Analysis (SOFA), a computational framework for multi-omics analysis. SOFA disentangles known from novel variation, and infers latent factors that give insights into diverse biological processes.

## Introduction

Using multiple “omic” measurement technologies on the same large set of biological specimens or samples is a fundamental design principle for studying biological variation^1–4^. With thousands or millions of features measured, the manifold hypothesis posits that the data concentrate along an (unknown) low-dimensional manifold inside the high-dimensional data space. Biological variation then corresponds to different positions on that manifold. Empirically, it has been found that useful coordinates on that manifold—what we might call interesting axes of biological variation—can be constructed by linear summary statistics. For instance, in principal component analysis (PCA) or its extension to factor analysis, each principal axis, or factor, is a linear combination of features, and this (weighted) averaging has the effect of reducing noise compared to looking at variation only by measuring individual features. Since typically a relatively small number of top principal axes or factors is considered, such approaches may also be viewed as dimension reduction techniques.

To take into account the multimodal structure of data, there are extensions of these methods, including canonical correlation analysis (CCA)^5^, group factor analysis (GFA)^6^, multi-omics factor analysis (MOFA)^7^, iCLuster^8^, and MuVI^9^.

Applied to data, these methods return factors that, typically, fall into two categories: unsurprising and surprising. Unsurprising factors include those that align with known covariates (e.g., in the case of tissue biopsies, disease diagnosis, age or sex of the patient; in the case of environmental samples, geographical sampling location), or to technical artefacts^10^ (batch effects) associated with known experimental metadata. Surprising factors have no such easily identifiable explanation and are candidates for scientific novelty. The above-mentioned methods are unsupervised, in the sense that they do not use such already known information, and users are required to make their surprisingness considerations post-hoc^11^, in a process that is informal and error-prone.

Here, we present Semi-supervised Omics Factor Analysis (SOFA), a probabilistic factor model that jointly models multi-modal omics data and sample-level information (hereafter: guiding variables). SOFA approximates the data with a low-rank matrix decomposition and partitions the latent factors into guided factors, each associated with a guiding variable, and unguided factors. In this way, already expected biological and technical drivers of variation can be regressed out from the latent space, making the interpretation of the remaining “surprising” factors simpler and potentially more fruitful.

Our model takes inspiration from prior work in the unimodal setting. Principal component regression (PCR) uses PCA factors as covariates in a regression model to predict guiding variables^12^. However, PCR does not guarantee that its covariates are relevant, since for their construction, it only considers their covariance structure, but not the guiding variables. Partial least squares (PLS) simultaneously decomposes a high-dimensional set of predictors and guiding variables, aiming to find factors that capture the maximum covariance between the two^13,14^. SPEAR^15^ and DIABLO^16^ enable the incorporation of guiding variables of interest in the multimodal setting. Like PLS, these methods primarily uncover “unsurprising” factors that align with the guiding variables, rather than explicitly identifying “surprising” or unexpected factors.

We present four applications of SOFA that highlight its broad utility: to the pan-gynecologic cohort of The Cancer Genome Atlas (TCGA)^17^, to the cancer dependency map^18,19^ (DepMap), to single-cell multi-omic data of microglial cells^20^, and to a collection of data sets of acute and chronic human heart failure^21–26^. We provide the SOFA method as an open-source Python package along with comprehensive tutorials.

## Results

### The SOFA model

**Fig. 1:**
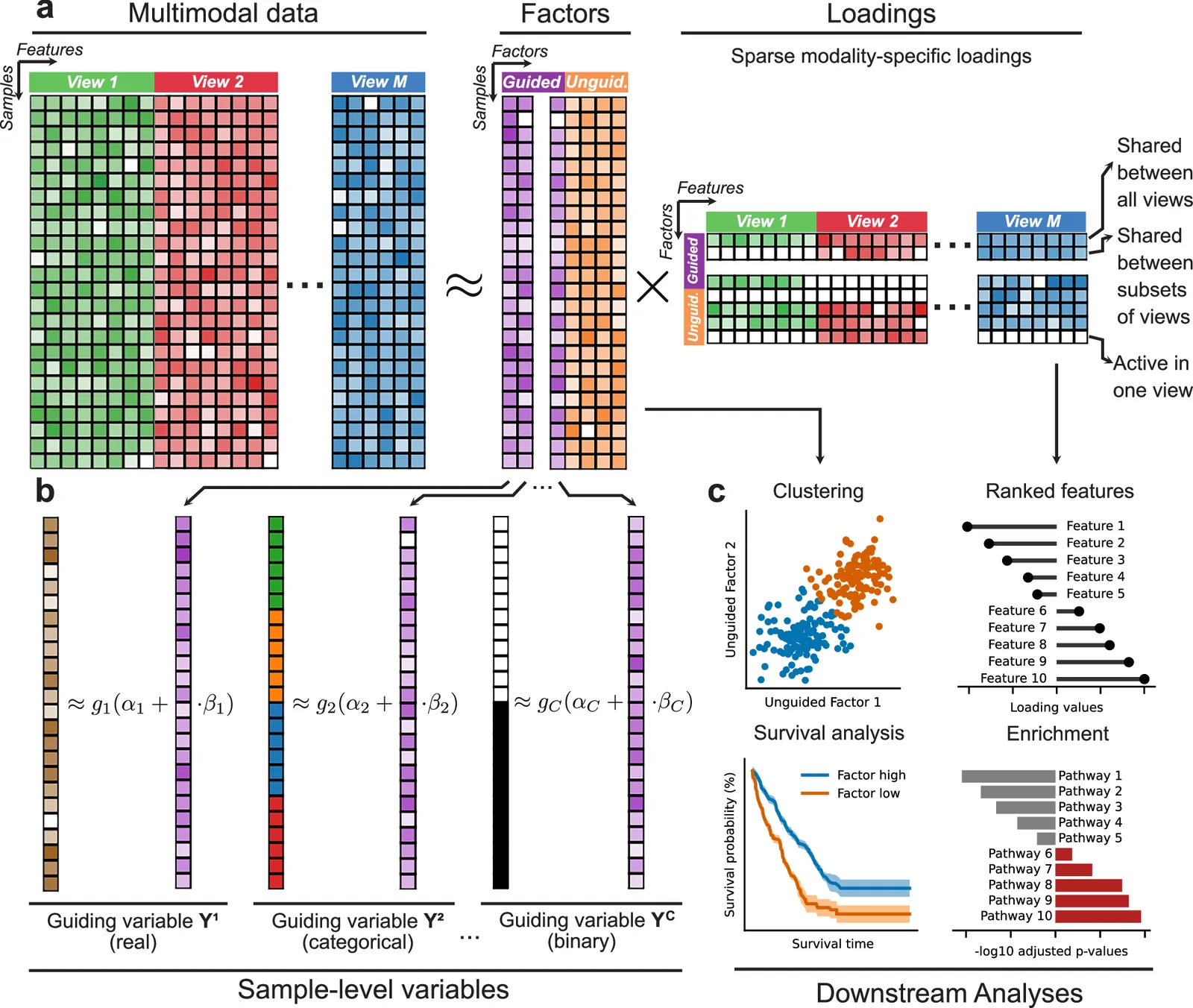
Overview of the SOFA model and downstream analyses. **a**: The SOFA model. Input data modalities X are decomposed into shared latent factors Z and modality-specific feature loadings W. A hierarchical horseshoe prior shrinks the loading weights on the view, factor and feature level. This leads to factors shared between all views, factors active in one view and factors shared between subsets of views. **b:** Guided factors are linked to the guide variables. **c:** Factors not linked to a guide variable capture potentially novel sources of variation. Factors and loadings can be used for various sample- and feature-level downstream analyses.

The input to SOFA is a set of numeric data matrices containing multi-omic measurements from the same or mostly overlapping samples, and one or more covariates for each sample. The covariates can be continuous, binary or categorical and contain information that is known or suspected to be important for driving biological or technical variation between the samples. We also call these covariates *sample-level guiding variables*, and the different types of omics data *views*. SOFA models each omics view with a Gaussian likelihood and therefore expects omics data appropriately normalized and transformed. For the guiding variables users can choose between Bernoulli (binary), Gaussian (continuous) and Categorical (categorical) likelihoods. From this input, SOFA extracts a lower-dimensional representation comprising a shared factor matrix and modality-specific loading matrices (Fig. 1a). SOFA partitions the latent factors into guided factors each associated with a guiding variable, and unguided factors (Fig. 1b). The loading weights of each factor represent, for each view, the molecular features that it depends on, and can usually be interpreted in terms of biological processes, gene expression programs, modes of regulation, and the like (Methods, Fig. 1c). The factor matrix positions each sample in the latent space spanned by the factors, and each sample’s coordinates along the various factors can be interpreted as a degree of activity of a factor in the sample, similarly as in PCA or MOFA.

SOFA is a hierarchical Bayesian factor model, and we ensured scalability of its inference to large multi-omic data sets by employing stochastic variational inference (Methods). Our inference method for SOFA employs a sparsity-inducing prior^27^, which pulls the loading weights of some factors for some modalities to zero if the data are not pulling them elsewhere strongly enough. As a result, we can distinguish between variation that is commonly shared across modalities and variation that is present in only one or a few modalities.

### SOFA identifies cancer type-independent immune infiltration vs proliferation axis in the TCGA pan-gynecologic data set

We applied SOFA to a pan-gynecologic cancer multi-omic data set of the Cancer Genome Atlas (TCGA) project, which profiled the transcriptome (mRNA), proteome, methylome, and miRNA of 2599 samples from five different cancers^17,28–31^. Additionally, the study includes data about mutations, metadata, and clinical endpoints, progression-free interval (PFI) and overall survival (OS).

**Fig. 2:**
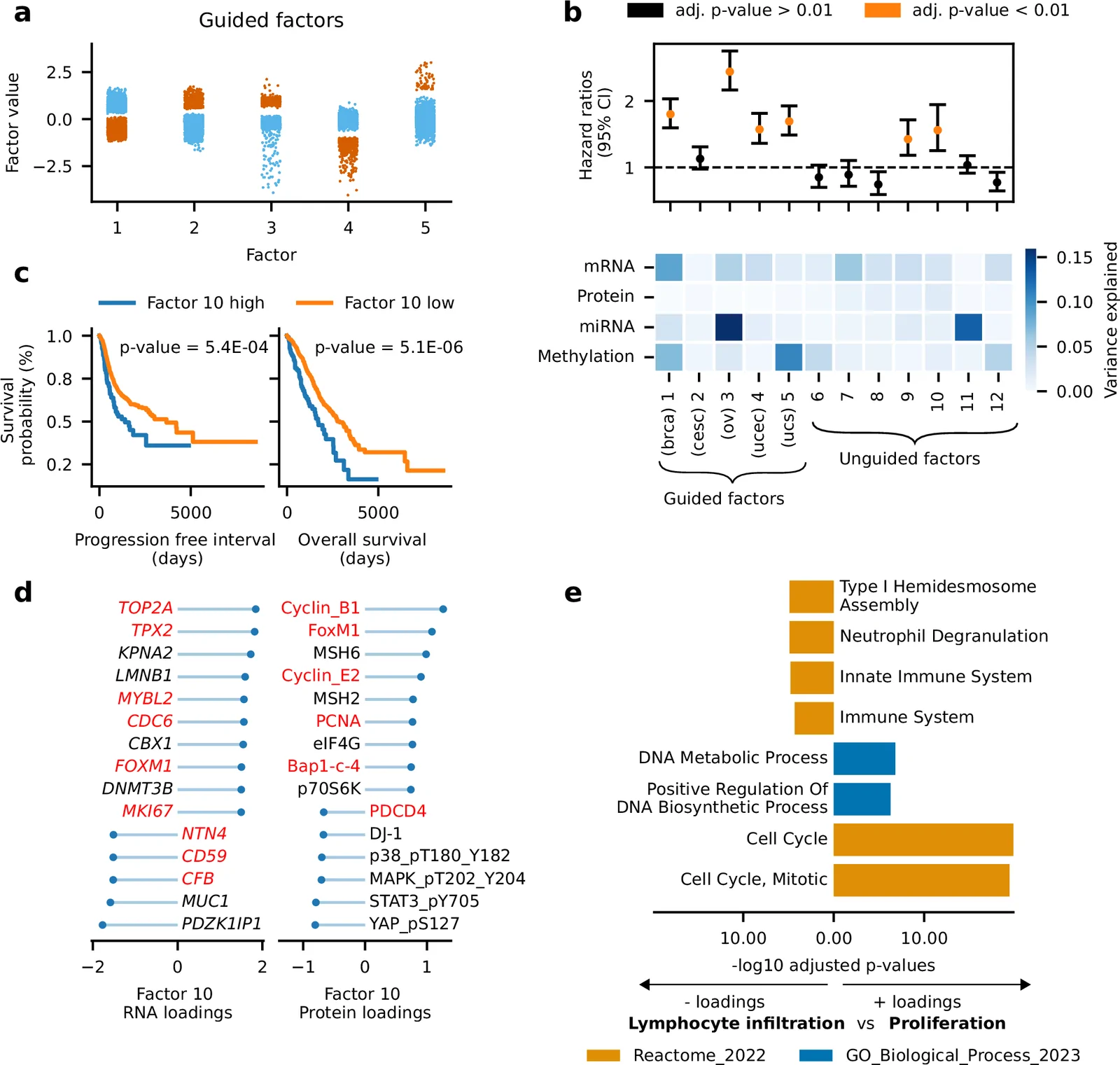
SOFA factors are significantly associated with survival and capture immune infiltration vs proliferation axis. **a:** Distributions of guided factors colored by their guiding variables (orange). **b**: Top: Hazard ratios from univariate Cox regression for each factor on overall survival (*n* = 2582). Hazard ratios > 1 indicate that positive factor values are associated with higher risk. Error bars indicate the 95% confidence interval. Hazard ratios are colored if adjusted *p* values are lower than 0.01 (log-likelihood ratio test). *P* values were adjusted for multiple testing using the Benjamini-Hochberg (BH) method. Adjusted *p* values were: 1.31e-20 (Factor 1), 0.35 (Factor 2), 4.5e-47 (Factor 3), 5.66e-9 (Factor 4), 1.15e-14 (Factor 5), 0.35 (Factor 6), 0.49 (Factor 7), 0.055 (Factor 8), 0.001 (Factor 9), 0.0006 (Factor 10), 0.58 (Factor 11), 0.031 (Factor 12). Bottom: Variance explained by each factor for each modality. **c**: Kaplan-Meier curves showing progression-free survival (left) and overall survival (right) for samples with factor values <50th and >95th percentiles of Factor 10. *P* values were computed using a two-sided log-rank test (*n* = 2582). **d**: Features with the highest absolute loading weights for factor 10 in transcriptomics (left) and proteomics loadings (right). **e**: Gene set overrepresentation analysis (one-sided hypergeometric test) for the transcriptomics features with the highest absolute loadings of factor 10. The -log10 adjusted *p* values are multiplied by the sign of the loadings to reflect the direction of the association with the factor. Cancer types shown: brca: breast invasive carcinoma; cesc: Cervical squamous cell carcinoma and endocervical adenocarcinoma; ov: Ovarian serous cystadenocarcinoma; ucec: Uterine Corpus Endometrial Carcinoma; ucs: Uterine Carcinosarcoma. Source data are provided as a Source Data file.

We performed a SOFA rank-12 decomposition, using five guided factors with binary labels for each cancer type and seven unguided factors. Guided factors showed a clear separation based on the binary cancer type labels (Fig. 2a). We then verified that the unguided factors did not capture cancer type-related variance by computing Adjusted Rand Index (ARI) scores to assess clustering alignment with cancer labels (Figure S2e, Methods). This analysis showed that the set of unguided factors had the lowest alignment with cancer type labels, suggesting that the guided factors mostly explained the cancer-associated variability. Of note, ARI scores for the unguided SOFA decompositions at both rank-7 and rank-12 exhibited lower cluster alignment in comparison to the guided factorization, indicating that factor guidance helped disentangle cancer-associated variability (Figure. S2a, c, e).

To evaluate the importance of each factor, we calculated the fraction of variance explained by each factor across the input modalities (Fig. 2b). For example, Factor 3 explained the highest fraction of variance in the miRNA data, consistent with previous research suggesting miRNA expression is critical in ovarian cancers and may serve as a biomarker^32,33^. Factor 1, representative for breast cancers, showed breast cancer-associated proteins AR^34^, PR^35^ and ER-alpha^36^ among the largest proteomics loading weights (Figure S2f). Factor 3 (ovarian serous cystadenocarcinoma) featured *BCAM*^37^*, MALAT1*^38^ *and WT1*^39^ among the highest transcriptomics loading weights (Figure S2g).

We then explored the SOFA factors for potential biological relevance by testing them for association with disease outcome, using univariate Cox regression of each factor on overall survival (OS, Fig. 2b). Notably, Factor 3, linked to ovarian serous cystadenocarcinoma, was significantly linked to poor survival (Hazard Ratio (HR) = 2.44 95% CI: 2.17–2.75). Two out of seven unguided factors showed significant associations with OS, suggesting that most of these factors do not strongly influence survival outcomes.

Among the unguided factors predictive of survival, we further investigated Factor 10, which had the highest HR among the unguided Factors (HR = 1.56 95% CI: 1.25–1.94, Fig. 2b). Stratifying samples by Factor 10 values, followed by non-parametric survival curve estimation, revealed substantial differences in median survival times between groups (OS: Median survival time 1701 vs. 2888 days, PFI: 1396 vs 3669 days, Fig. 2c). High values of Factor 10 were associated with increased expression of proliferation-related genes and oncogenic pathways, while low values were enriched for immune-related signatures, suggesting an antagonism between tumor proliferation and immune activity. Negative loadings of Factor 10 featured *NTN4*, *CD59* and *CFB*, and proteins including PDCD4, indicative of complement system activation and lymphocyte infiltration^40–43^ (Fig. 2d). In contrast, positive loadings included proliferation markers *TOP2A* and *TPX2*, as well as Cyclin B1, FoxM1^44–47^, suggesting a signature of increased proliferation. This was further corroborated by performing a gene set overrepresentation analysis on the top transcriptomics loadings. Negative loadings were associated with immune system related gene sets, while those with positive loadings were enriched in cell cycle and proliferation related terms (Fig. 2e). Finally, we note that Factor 10 was not restricted to a single cancer type (Figure S2b, d), indicating that it captured biological signatures common to all cancers. Taken together, we found that the guided SOFA factorization delineated cancer type-specific variation from broader biological processes, such cell proliferation or immune cell infiltration, thereby facilitating their identification and subsequent downstream analyses.

### SOFA identifies EGFR dependency axis in pan-cancer dependency map, while accounting for known driver mutations and cell growth

Next, we analyzed a pan-cancer cell line data set of the Cancer Dependency Map (DepMap)^19,48^ together with proteomics data from the ProCan-DepMapSanger study^18^. The DepMap project aims to identify cancer vulnerabilities and drug targets across a diverse range of cancer types. The data set includes multi-omic data, encompassing transcriptomics^49^, proteomics^18^, and methylation^50^, as well as drug response profiles for 627 drugs^50^, and CRISPR-Cas9 gene essentiality scores^48^ for 17,485 genes for 949 cancer cell lines across 26 different tissues.

To identify CRISPR-Cas9 gene essentiality scores associated with multi-omic features, we performed a rank-20 SOFA decomposition to the DepMap data, while accounting for potential/known drivers of variation such as growth rate, microsatellite instability (MSI) status, *BRAF*, *TP53*, and *PIK3CA* mutation and hematopoietic lineage. Although CRISPR-Cas9 essentiality scores could technically be included as an additional view in the SOFA decomposition, we chose to exclude them to enable independent testing of factor associations with these scores.

**Fig. 3:**
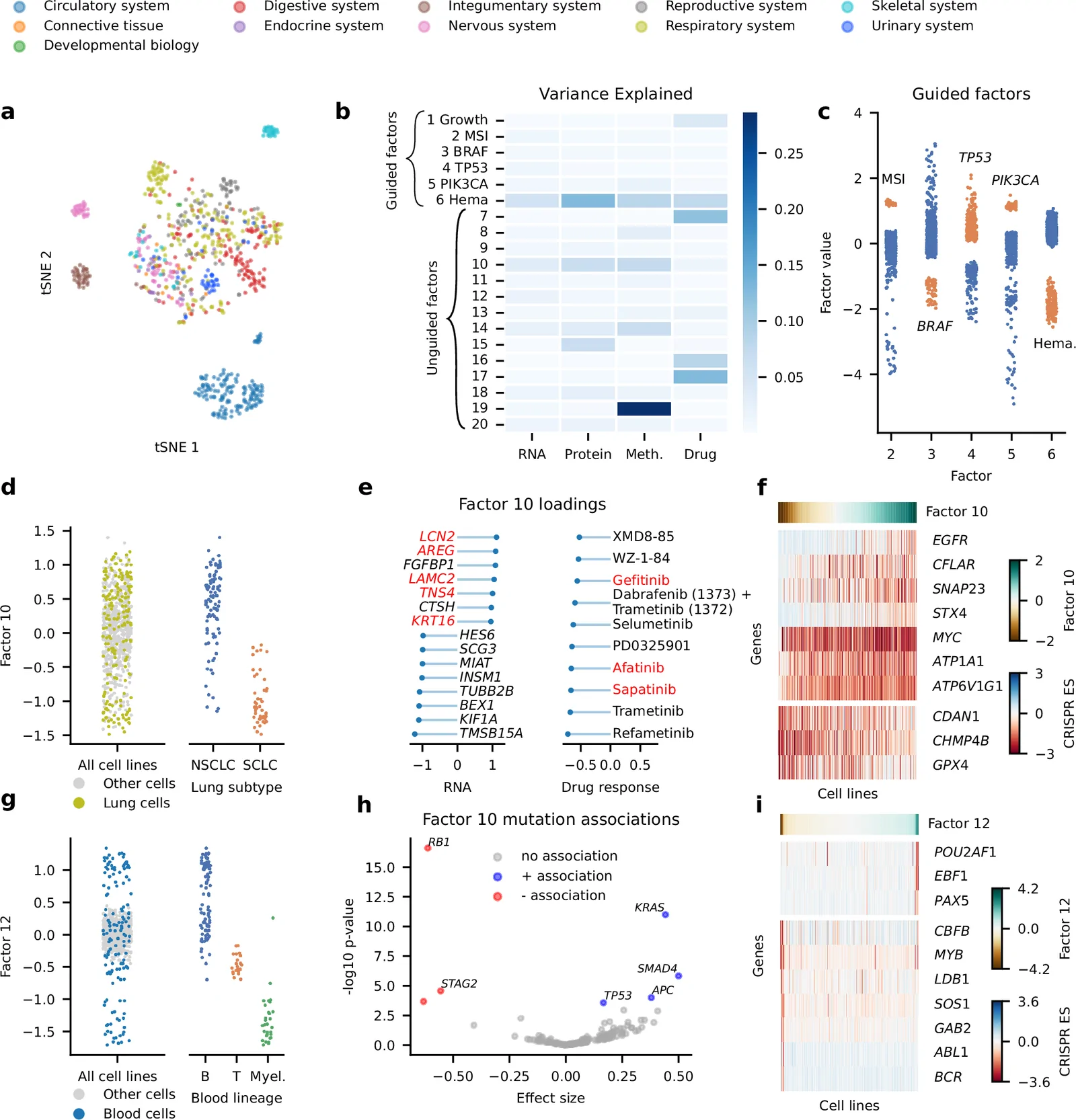
SOFA factors capture lineage subtypes and EGFR dependency axis in DepMap. **a:** tSNE of all SOFA factors, colored by cancer types. **b**: Variance explained by each factor for each modality. **c**: Distributions of guided factors colored by their guiding variables (orange). **d**: Distribution of Factor 10 cell lines from the respiratory system are colored (left). Distribution of Factor 10 for cell lines from non-small cell lung cancer (NSCLC) and small cell lung cancer (SCLC) (right). **e**: Highest absolute transcriptomics (left) and drug response (right) loadings of Factor 10. **f**: Heatmap showing CRISPR essentiality scores (ES) of top 10 genes significantly associated with Factor 10, tested using a linear model (two-sided *t*-test of the slope coefficient; *n* = 778). The cell lines are ordered based on their values of Factor 10. **g**: Distribution of Factor 12, cell lines from the circulatory system are colored (left). Distribution of Factor 12 for B-cell, T-cell and myeloid cell lines (right). **h**: Volcano plot of associations between Factor 10 and mutations, tested using a two-sided two-sample *t*-test (*n* = 778). Effect sizes are the differences in means between mutated and unmutated samples. BH-adjusted *p* values < 0.05 are highlighted in color. Genes colored in blue are significantly associated with higher and genes colored in red with lower factor values. **i**: Heatmap showing CRISPR essentiality scores (ES) of top 10 genes significantly associated with Factor 12, tested using a linear model (two-sided *t*-test of the slope coefficient; *n* = 778). The cell lines are ordered based on their values of Factor 12. Source data are provided as a Source Data file.

A t-SNE of all the inferred factors showed that they captured the differences between cancer types (Fig. 3a). Further, we confirmed successful factor guidance by asserting that SOFA extracted high or low factor values in a variable-dependent manner (Fig. 3c). We then focused on the guided factors and observed that Factor 6, consistent with previous studies identifying hematopoietic lineage as a major driver of variation in cell lines^18^, explained the largest fraction of variance (34%) across all data modalities (Fig. 3b). Factor 1, guided by growth rate, mostly explained variance in drug response (Fig. 3b), which is expected as the natural logarithm of the half maximal inhibitory concentration (lnIC50) drug response is derived from the growth rate of the cell line. We then subjected the loadings of guided factors to gene set overrepresentation analysis. Expectedly, Factor 3, guided with labels for BRAF mutation, a common mutation in skin cancer^51^, was associated with skin cancer related gene sets (Figure S3c). The loadings of Factor 6, guided with hematopoietic lineage, displayed gene sets related to the immune system (Figure S3d). Statistical testing revealed that Factors 3 and 4 were significantly associated with CRISPR-Cas9 essentiality scores of BRAF and TP53, the mutations used to guide these factors (Figure. S3e, f, Methods).

Among the unguided factors, Factor 10 had the highest values in colorectal and lung cancer cell lines (Figure. S3a). A closer examination revealed that this factor delineated non-small cell lung cancer (NSCLC) from small cell lung cancer (SCLC) (Fig. 3d). Top-ranking genes featured regulators of the epidermal growth factor receptor (EGFR), including *LCN2, AREG, LAMC2, TNS4* and *KRT16*^52–56^ (Fig. 3e). Similarly, the lowest loadings for drug response lnIC50 values, indicating sensitivity, were EGFR targeting drugs Gefitinib, Afatinib and Sapatinib (Fig. 3e). To confirm the association between the EGFR pathway and Factor 10, we tested for associations with CRISPR-Cas9 essentiality scores, indeed EGFR was a top-ranking association (Fig. 3f). Further, high Factor 10 values were associated with cell lines harboring *EGFR*, *TP53*, *APC*, *SMAD* and *KRAS* mutations, which were previously described to play a role in progression of colorectal cancer^57,58^ (Fig. 3h).

Factor 12 separated B cells, T cells, and myeloid cells within the hematopoietic lineage (Figs. 3g and S3b). Correlating Factor 12 with CRISPR-Cas9 essentiality scores showed that positive factor values (B-cells) were significantly associated with B-cell markers *EBF1*, *PAX5* and *MEF2B*^59,60^, while negative values (myeloid cells) were significantly linked to myeloid markers *MYB* and *CBFB*^61,62^ (Fig. 3i). In summary, by leveraging multi-omic data together with CRISPR-Cas9 essentiality scores, we identified an unguided factor linked to the EGFR pathway, along with another factor that explained the difference between hematopoietic cell types.

### SOFA disentangles between and within cell type variation in a single-cell multi-omic data set of the human developing cerebral cortex

We applied SOFA to infer 15 factors from data of a single-cell multi-omic experiment^20^, where the authors simultaneously profiled the transcriptome (RNA) and chromatin accessibility (ATAC) of 45,549 single cells of the human cerebral cortex at six different developmental stages from 12 different donors. The authors identified 13 different cell types in the data.

We assumed that guiding a subset of SOFA factors with cell type labels could help reveal variations within cell types through the unguided factors. To test this, we conducted a rank-15 SOFA decomposition on the single-cell multi-omic data set, guiding the first 13 factors with binary cell type labels while leaving the remaining two factors unguided to capture sources of variation within cell types.

**Fig. 4:**
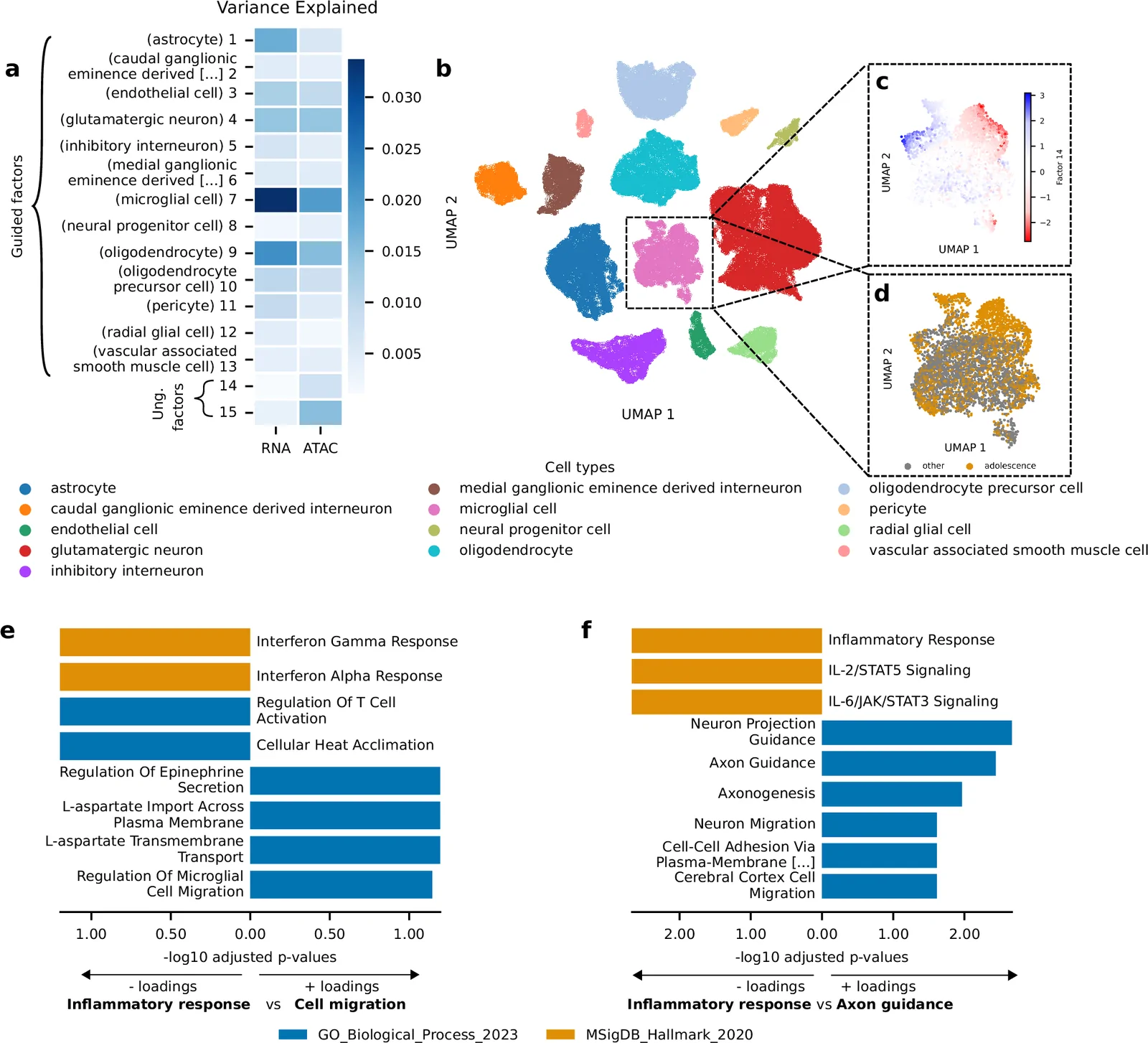
SOFA disentangles between and within cell type variation. **a**: Variance explained in RNA and ATAC data modality by each factor. **b**: UMAP based on all factors colored by cell type. **c**: UMAP of microglial cells colored by Factor 14. **d**: UMAP of microglial cells colored by adolescence age group. **e**: Gene set overrepresentation analysis (one-sided hypergeometric test) for the transcriptomics features with the highest absolute loadings of factor 14. The -log10 adjusted *p* values are multiplied by the sign of the loadings to reflect the direction of the association with the factor. **f**: Gene set overrepresentation analysis (one-sided hypergeometric test) for the ATAC features with the highest absolute loadings of factor 14. The -log10 adjusted *p* values are multiplied by the sign of the loadings to reflect the direction of the association with the factor. Source data are provided as a Source Data file.

Figure 4a shows the fraction of variance that each factor explained in the RNA and ATAC modalities, with factors guided by microglial and oligodendrocyte labels explaining most variance. The UMAP embedding of all factors in Fig. 4b showed that the cells cluster by cell type clearly separating the different cell types.

First, we checked whether the loadings of the guided factors picked up biological signals related to their guiding covariate. Indeed, the guided factors separated cells based on their guiding cell type labels (Figure S4a). The top loadings of the guided factors were cell-type-specific markers of their respective guiding cell type label. Highest transcriptomics loadings of Factor 1 (astrocytes) included astrocyte-specific marker genes such as *SLC1A2*, SLC1A3^63^ and *GLUL*^64^. Factor 7 (microglial cells) captured microglia markers *CSF1R*^65^, *TYROBP*^66^ and *CX3CR1*^67^. Factor 9 (oligodendrocytes) had *MBP*^68^*, MOBP*^69^ and *PLP1*^70^ among the top transcriptomics loadings (Figure S4b–d).

When further inspecting the unguided factors, we found that Factor 14 captured variation within microglial cells and was only active in a subset of microglial cells with relatively high and low factor values (Fig. 4c). Gene set overrepresentation analysis on the transcriptomics and ATAC loadings of Factor 14, revealed that the positive loadings are related to microglial migration and transport processes, while the negative loadings play a role in inflammatory response and interleukin signaling (Fig. 4e, f). Previous studies using single-cell RNA-seq data from microglia indicated that there is a diverse range of microglial subtypes^71^, including populations expressing genes related to inflammatory response^72^ and migration^71^. Furthermore, microglia play a critical role in brain development^73^, especially during adolescence^74^ and Factor 14 was mostly active in cells from the adolescence age group (Fig. 4c, d).

This shows that SOFA is also capable of finding biologically interesting patterns, such as within cell type variation, in single-cell multi-omic data sets.

### Comparison of SOFA and MOFA in the analysis of heart failure single-cell data

We compared SOFA’s guided factor analysis approach to MOFA’s unsupervised one to evaluate whether SOFA enhances detection of covariate-associated low-variance signals, while preserving the integrity of factors representing dominant variability. We applied both methods in the context of multicellular integration of single-cell RNA-Seq data, which aims at inferring coordinated gene expression programs between cell types in tissues, across a previously curated collection of five data sets of acute and chronic human heart failure^21–26^. This collection of data sets includes single-cell RNA-Seq data of seven cell types from chronic human heart failure cases (HF) and controls (NF), along with covariates including age, sex, and heart failure status, a binary variable indicating patients with any heart disease etiology that required heart transplantation (Figure S5a, b). To apply SOFA and MOFA, we pseudo-bulked the gene expression of each cell type and represented them as separate views (Figure S5c). This enables the inferred factors to capture gene expression coordination across cell types—referred to as multicellular programs—which could then be associated with patient-level covariates. Heart failure was expected to be the primary source of variability in these data sets^21^. However, given the smaller cohort sizes compared to our previous analysis examples (maximum size = 79), low-variance covariates like age and sex might be challenging to detect using an unsupervised model. We fitted a MOFA model and three SOFA models: one guided by age and sex, another that included guidance from both age and sex along with heart failure binary status, and a model only guided by HF. This approach allowed us to assess whether SOFA could identify factors associated with less dominant covariates and evaluate the impact of factor guidance on the detection of unguided factors representing major sources of variability, in this case, heart failure. We observed that both methods recovered a comparable fraction of the variance associated with heart failure and age. However, SOFA models recovered a greater fraction of variance (mean = 3.65% SOFA HF guided; mean = 3.7% SOFA HF unguided) associated with sex across data sets compared to MOFA (mean = 0.43%), which showed near-zero values in all but one data set (Figure S5d). To see whether the differences between SOFA and MOFA in explaining sex-related variance were due to differences in regularization, we compared them with SOFA models guided only by HF. We found that these simpler SOFA models failed to capture factors with weak sex signals and explained less HF-related variance than the fully guided SOFA models in three of the five studies.

Next, we compared the fully guided SOFA model with a fully unsupervised MuVI model, which, unlike MOFA, uses a horseshoe prior as its regularization strategy. We found no factor from the MuVI model associated with sex across studies.

These comparisons suggest that the improved capture of low-signal features is driven by SOFA’s guidance, rather than the differences in regularization between models. To compare the guidance strategy of SOFA to the ones used by alternative models that are able to introduce prior knowledge to factor reconstruction, we fitted two additional models using SPEAR^15^ and MuVI^9^. SPEAR and MuVI models used sex labels and genes linked to the Y chromosome, respectively, to guide factor reconstruction. When comparing the guided-SOFA models to the SPEAR models across all data sets, we found that SOFA consistently recovered a greater fraction of variance associated with sex and age (one-sided *t* test, adjusted *p* value < 0.05). Additionally, SOFA recovered a greater fraction of variance associated with HF in 2 out of 5 studies, performing worse in only one. Compared to the sex-guided MuVI model, SOFA recovered greater variance fractions associated with sex in three out of five studies (one-sided *t*-test, adjusted *p* value < 0.05), with the two remaining ones performing similarly. SOFA, in addition, recovered a greater fraction of variance associated with HF in 3 out of 5 studies, performing worse in only one.

To assess whether guiding SOFA with known covariates would hamper the unguided factors to capture the main sources of variability—here heart failure—, we correlated the heart failure-associated factors between the MOFA model and the SOFA model not guided by heart failure. The mean Spearman correlation of 0.81 and 0.870 at both the factor score and gene loading levels indicated minimal influence of factor guidance on these unguided factors. Next, we evaluated whether the loadings of the heart failure and sex-associated factors across the three models captured similar results to the ones expected from differential expression analyses. First, we used DEseq2 models to identify differentially expressed genes between failing and non-failing hearts, and males and females, across cell-types for each data set independently (adjusted *p* value < 0.05). Then we sampled an identical number of genes from the gene loadings ranked by their absolute value. We observed higher recall for heart failure of the SOFA model guided with heart failure (mean = 66.2%) compared to the other two models (mean=55.05% SOFA HF unguided; mean = 55.25% MOFA) (Figure S5e). For the sex-associated factors, we observed a mean recall of 55.84% in the SOFA models.

To assess whether SOFA’s factor guidance identifies subspaces distinct from those of MOFA, we compared the RV coefficients of unguided and guided SOFA models to those of a MOFA model. As shown in Figure. S6e, the guided SOFA model exhibited lower RV coefficients than the unguided model, indicating reduced similarity to the MOFA subspaces.

Together, these results highlight that guided SOFA factors can enhance the detection of gene expression profiles associated with low-variance covariates like sex while preserving the ability to infer major sources of variability, providing a flexible alternative to fully unsupervised models.

## Discussion

SOFA is a fully interpretable probabilistic framework for the analysis of multi-omic data.

It is based on group factor analysis methods such as GFA and MOFA^6,7^ and decomposes multi-omic data into latent factors that capture shared or modality-specific variance across multiple omics modalities. In contrast to existing latent factor models, it enables the incorporation of a priori known (or suspected) drivers of variation. The drivers of variation can be covariates such as mutations, pathways, cancer or cell types. While the guided factors explain variation in the molecular omics data that is driven by the guiding variables, the unguided factors can capture the remaining variation, potentially leading to novel biological insights. While fully supervised matrix decomposition methods such as DIABLO and SPEAR infer latent factors that are predictive of a response (or guiding) variable of interest, SOFA explicitly partitions the latent factors into supervised (or guided) factors and unsupervised (unguided) factors.

Previously reported analysis approaches to such data either involved manual post-hoc annotation of factors or splitting of the data into subsets of cell types, tissues, etc., to identify subset specific factors^18,21,75^. SOFA offers a potentially easier and more straightforward workflow by employing the complete data and the guiding variables in a single end-to-end analysis.

The versatility of SOFA was showcased through four examples of exploratory data analysis. Analysis of the pan-gynecologic cohort of the TCGA^17^ revealed a biological stratification predictive of survival and independent of cancer type. In a second example, we identified a latent factor related to EGFR cancer dependency enriched in lung and colorectal cancer samples in a pan-cancer cell line analysis of the DepMap^18,19^. Applying SOFA to a single-cell multi-omic data set from cells of the developing human cerebral cortex^20^, we show that SOFA can account for inter-cell type heterogeneity, while discovering factors explaining intra-cell type heterogeneity. We compared SOFA, MOFA, MuVI and SPEAR in an integrative analysis of five data sets of acute and chronic human heart failure. MOFA, due to its unsupervised nature, concentrates on high-variance signals in the data. This can lead to low-variance signals not being picked up by MOFA. SOFA’s guided approach can help to extract such signals if they are associated with a known covariate. SPEAR can include guiding variables, but the guiding variables potentially influence the whole latent space and are not assigned to specific factors, effectively separating known variance from unknown. MuVI uses a similar regularization strategy to SOFA (horseshoe priors), but, in contrast to SOFA, it answers a different question: it integrates prior feature groupings through structured sparsity priors and thus aligns latent factors with predefined biological sets to enhance interpretability, but it does not have the more explicit covariate supervision that SOFA provides.

SOFA is a tool for exploratory analysis of multi-omic data sets. It identifies sets of features (e.g., representing genes and gene products) that covary across multiple modalities. It is aimed to generate hypotheses that will be validated with independent test data or follow-up experiments. SOFA’s unguided factors are comparable to factors from other factor analysis models such as MOFA and is thus a technique for exploratory data analysis. To discern unguided factors that pick up technical variation from biologically relevant factors, users need to inspect their loadings and correlate them with additional knowledge (e.g., disease subgroups). It may also be instructive to see whether they might reflect interactions between other factors or residuals from non-linear relationships. In practice, factors should be validated in independent data sets or by a mechanistic study to instil confidence in their generalizability. Penalized multivariate regression on the validation can be used as outlined in ref. ^11^ to predict the factors of interest.

SOFA is a linear method, which facilitates interpretation, but would be a limitation if there are important phenomena that are only evident through non-linear functions of the features. If there is reason to assume that interactions between two or more guiding variables play a role, then users can simply augment the set of guiding variables and define additional ones, e.g., by multiplying two guiding variables to model an interaction between the two variables, or indeed using any other non-linear function of two or more variables. Examples may include gene-drug interactions such as TP53 mutation and sensitivity to Nutlin, or BRAF V600E mutation and sensitivity to vemurafenib.

On the other hand, if such interactions are not on the analysts’ mind upfront, they may fit a SOFA model with a given set of guiding variables and then find an unsupervised factor that matches a non-linear function of one or several guiding variables. In such cases, they may take this finding forward directly or define a new guiding variable that expresses this interaction and refit SOFA to find further, perhaps yet more subtle, latent factors.

Furthermore, inclusion of too many guiding variables can degrade the capacity of SOFA to discover latent factors. In particular, this can happen if one or several guiding variables are approximately (multi-)collinear with one or several latent factors of interest. In such cases, lack of power from finite data size can lead to masking of the latent factors^76^. Analysts can explore such situations by repeating SOFA fits with and without certain guiding variables and then decide based on domain knowledge, or other practical considerations which of the guiding or latent (multi-)collinear variables are the most relevant or suitable ones for modeling the data at hand. SOFA can also be applied if no guiding variables are apparent, in which case it effectively reduces to an unsupervised factor model, similar—up to regularization and symmetries—to MOFA. In a subsequent step, users may study the discovered latent factors and decide to identify—by correlation—one or several of them with previously overlooked, explicit variables, and then make a renewed call to SOFA, now with these explicit variables as guiding variables. Similarly, users may first use other unsupervised methods to discover important guiding variables and then use SOFA to do a factor analysis on the remaining variation.

The current implementation of SOFA considers each omics view with a Gaussian likelihood. For highly heteroskedastic data, such as, for instance, RNA-seq, appropriate preprocessing and transformation is required.

In summary, SOFA is able to discover novel axes of variation while accounting for previously known variation. It is implemented using the probabilistic programming framework Pyro^77^, is available as a Python package including documentation and analysis examples, and its processing speed makes it amenable to interactive analysis on data sets such those shown here.

## Methods

### The SOFA model

SOFA jointly decomposes a multimodal data set ${{{\bf{X}}}}^{m}\in {{\mathbb{R}}}^{N\times {D}^{m}}$ with *m* = *1,…,M* modalities for *i* = *1,…,N* samples and *j* = *1,…,D*^*m*^ features, and a set of continuous or categorical sample-level covariates (hereafter guiding variables) **Y**^1^,…, **Y**^C^, where ${{{\bf{Y}}}}^{{{\boldsymbol{c}}}}\in {{\mathbb{R}}}^{N}$ with *c* = *1,…,C* guiding variables, into a lower dimensional shared latent factor matrix and modality-specific loading matrices. We model the entries *x*_*ij*_^*m*^ of **X**^**m**^ as normally distributed, given an unobserved (latent) mean *µ*_ij_^m^ and a feature-specific scale parameter *σ*_j_^m^, and the entries *y*_*i*_^*c*^ of **Y**^c^ with a mean/location parameter *ν*_i_^c^ and an optional scale parameter *κ*^c^

1 $${x}_{{ij}}^{m} \sim N({\mu }_{{ij}}^{m},{\sigma }_{j}^{m})\;\;{{\rm{and}}}\;\;{y}_{{{\rm{i}}}}^{{{\rm{c}}}} \sim {p}^{c}({\nu }_{i}^{c},{\kappa }^{c})$$

where *p*^*c*^ denotes an appropriate distribution (we currently support Bernoulli for binary data, Categorical for categorical data and Normal for real valued data) for the *c*th guiding variable. We express *µ*_ij_^m^ and *ν*_i_^c^

2 $${\mu }_{{{\rm{ij}}}}^{{{\rm{m}}}}={\Sigma }_{k=1}^{K}{z}_{{ik}}{w}_{{kj}}^{m}\;\;{{\rm{and}}}\;\;{\nu }_{i}^{c}={g}_{c}({\alpha }_{c}+{z}_{{ic}}{\beta }_{c})$$

where ${w}_{{kj}}^{m}$ represents the entries of the *m*th loading weight matrix ${{{\bf{W}}}}^{m}\in {{\mathbb{R}}}^{K\times {D}^{m}}$ consisting of *k* = *1,…,K* components and *C* ≤ *K ≤ min(D*_*1*_*,…,D*_*M*_*)*, ${z}_{{ik}}$ the elements of a factor weight matrix ${{\bf{Z}}}\in {{\mathbb{R}}}^{N\times K}$ containing the coordinates of each sample with respect to ${{{\bf{W}}}}^{m}$, ${\alpha }_{c}$ and ${\beta }_{c}$ an offset and slope parameter and ${g}_{c}$ an appropriate inverse link function (see extended methods for the supported link functions) for the *c*th guiding variable. While there is a one-to-one relationship between guiding variables *Y*^*C*^ and inverse link functions ${g}_{c}$, their arguments depend, via ${\beta }_{c}$—depending on the user’s choice—‚ on multiple guided factors **Z**.

SOFA is formulated as a Bayesian probabilistic model and uses prior distributions on the model parameters (for a complete description of the model see the Supplementary Methods).

#### Interpretation of the factors

Analogous to the interpretation of factors in PCA, SOFA factors ordinate samples along a zero-centered axis, where samples with opposing signs exhibit contrasting phenotypes along the inferred axis of variation, and the absolute value of the factor indicates the strength of the phenotype. Importantly, SOFA partitions the factors of the low-rank decomposition into guided and unguided factors: the guided factors are linked to specific guiding variables, while the unguided factors capture global, yet unexplained, sources of variability in the data.

In Eq. (2), the guided features ${\beta }_{1,\ldots,}{\beta }_{C}$ combine linearly into the arguments of the inverse link functions ${g}_{1,\ldots,}{g}_{C}$. If the link function ${g}_{C}$ is the identity, the overall relationship between the mean parameter for the *c*-th guiding variable *ν*_i_^c^ and the latent guided factors is linear. Based on the chosen likelihood, non-linearities can be modeled with the link functions. Conversely, users may choose to transform some of the guiding variables *Y* ^*C*^, e.g., by multiplying two guiding variables to model an interaction term between them.

#### Interpretation of the loading weights

SOFA’s loading weights indicate the importance of each feature for its respective factor, thereby enabling the interpretation of SOFA factors. Loading weights close to zero indicate that a feature has little to no importance for the respective factor, while large magnitudes suggest strong relevance. The sign of the loading weight aligns with its corresponding factor, meaning that positive loading weights indicate higher feature levels in samples with positive factor values, and negative loading weights indicate higher feature levels in samples with negative factor values. We use a hierarchical horseshoe prior^27^ to induce sparsity in the loading weights, which tends to facilitate model interpretation in high-dimensional settings. However, we note that opting for the horseshoe was purely a choice of convenience, and other sparsity-inducing approaches may also lead to useful results.

### Parameter inference

SOFA is formulated as a Bayesian probabilistic model and uses black-box variational inference^78^ to infer the unobserved parameters. Black-box variational inference uses a simpler variational distribution to approximate the intractable posterior. It is a scalable technique that uses stochastic optimization to optimize the evidence lower bound (ELBO) with respect to the variational parameters of the variational distribution. The ELBO is optimized using stochastic variational inference by taking Monte Carlo samples from the variational distribution and computing unbiased but noisy gradients with respect to the variational parameters from the ELBO^78,79^. The ELBO can be used to assess convergence.

For the implementation of SOFA, we used the probabilistic programming framework Pyro^77^. If necessary, e.g. for single-cell data, we compute the gradients over subsamples of batches of the observations. Although variational approximations introduce bias, they allow efficient posterior inference on large data sets where fully Bayesian methods like MCMC are computationally infeasible.

### Model selection

The number of factors is an important hyperparameter of SOFA that needs to be determined by running the inference multiple times with a varying number of factors. We recommend looking at the Akaike (AIC) and Bayesian Information Criteria (BIC), which aim to balance goodness-of-fit with model complexity. Furthermore, factors typically become correlated if the decomposition rank is too high. Additionally, RMSE and variance explained can be used to assess good choices of the number of factors. However, in practice, similar to other unsupervised methods, such as clustering (number of clusters), PCA (number of principal components), it is not straightforward to choose the optimal number of factors solely based on model selection metrics, and we recommend looking for utility and interpretability of factors in terms of domain knowledge.

Finally, whether guiding variables potentially drive variation in the data needs to be tested in multiple inference runs.

### Choice of hyperparameters

SOFA allows users to specify the learning rate for model fitting. We recommend initializing the learning rate at 0.05 for the first 3000 epochs and gradually decreasing it to 0.001 over the final 3000 epochs. In practice, optimal learning rates and epoch numbers depend on the data set, and convergence can be monitored using the ELBO. To guide users, we implemented an early stopping criterion, which stops training when the improvement of the ELBO loss is lower than a user-chosen percentage.

To control the supervision strength of guiding variables, SOFA enables users to set a scaling factor for each variable. This factor adjusts the corresponding likelihood term, increasing emphasis on explaining the guiding variable. Higher scaling factors enforce stronger supervision, while a value of zero removes supervision entirely. We empirically found that a default value of 1% of the total number of features in the omics views leads to a good tradeoff between supervision and explained variance. This scaling factor was also used for all our analysis results.

### Simulations

To verify that SOFA is able to both infer factors that approximate the multi-omic data and simultaneously guide a subset of the factor, we simulated data from the generative model, inferred a varying number of latent factors and computed the root mean square error between the reconstructed and the simulated data.

Figure. S1a shows the RMSE for the simulated multi-omic data for an unguided and an SOFA model, where the first two factors were guided with simulated guide variables. The RMSE of the unguided model was slightly lower than that of the guided model. However, this was only the case for model fits with a lower number of factors than the simulated data. This indicates that if the number of latent factors is at least equal to the number of latent dimensions of the simulated data, SOFA is able to guide a subset of the factors without losing its ability to fit the multi-omic data. Furthermore, the simulated guide variables align with their respective guided factors (Figure. S1b, c).

Like MOFA, the SOFA model can be fit if subsets of the data are missing (e.g., if some views were only obtained for a subset of samples). The recovery of latent factors is fairly robust to missingness at random (Figure. S6a). However, we note that in practice, data missingness is often correlated and informative, and analysts need to mitigate missingness carefully.

To check whether multiple initializations of SOFA models find essentially the same subspace, we fitted 10 SOFA models with the TCGA^17,80^, DepMap^18,19,48^, single-cell multiome^20^ and a simulated data set and calculated the RV coefficient to one of the initializations. Figure. S6d shows that RV coefficients were close to 1 for all four data sets and indicates that SOFA yields consistent subspaces between different initializations.

We evaluated SOFA’s scalability using simulated data sets with three views, two guiding variables and 10 factors while increasing the number of samples. Figure. S6c shows that data sets with up to 150k samples (or cells) can be processed in ~30 min, requiring ~4 GB of memory on standard GPUs.

### Comparison of SOFA and MOFA in the analysis of heart failure single-cell data

To investigate how guiding a subset of factors influences factorization, we applied SOFA for multicellular integration^21^ to a collection of single-nucleus RNA-Seq data sets of acute and chronic human heart failure compiled in ref. ^22^. For each study, additionally, patient covariates such as age, sex and heart failure labels were available. Cell type annotations of each single-nucleus data were aligned to a unified ontology of seven cell-types: Cardiomyocytes, fibroblasts, pericytes, and endothelial, vascular smooth muscle, myeloid and lymphoid cells.

Pseudobulk expression profiles of each cell type per patient were used as input views for SOFA, as outlined in ref. ^21^. Briefly, pseudobulks for each cell type were included only if they contained at least 20 unique cells. Cell types present in fewer than 40% of the patients in a given data set were removed. At the gene level, genes expressed fewer than 20 times across 60% of the patients in a cell type were considered lowly expressed and filtered out. After this step, cell types with fewer than 50 expressed genes were discarded. Samples with less than 97% coverage of the filtered gene set were also excluded. Known background genes were removed, retaining only genes of interest for factor inference. Following these filtering steps, only cell types with at least 50 remaining genes were included in the final analysis.

We fit three models: a rank-10 MOFA model, a rank-10 SOFA model guided by age and sex, and a rank-10 SOFA model guided by age, sex, and heart failure. SOFA models were fitted with a total of 3500 steps and a learning rate of 0.01.

We identified factors associated with heart failure, sex, and age, and calculated the fraction of variance explained by these factors. This metric served as a proxy for the amount of variation in the omics data linked to heart failure, sex and age. Analysis of variance was used to associate factor scores with heart failure and sex, and Pearson correlation was used to test for association with age. When multiple factors associated with a covariate, we summed their fraction of variance explained.

To identify the most similar pair of heart failure-associated factors between the MOFA model and the SOFA model guided by age and sex in each study, we used absolute Spearman correlations of gene weights and factor scores, together with their fraction of explained variance. For each data set and model, we identified genes significantly associated with heart failure (adjusted *p* value < 0.05), using the R package DESeq2^81^ with default parameters to filter out lowly expressed genes. We selected the number of significant genes among the highly ranked genes in the model by using their absolute factor loading as the basis for ranking. We then calculated recall, defined as the percentage of loadings that included these expected genes in the right regulatory direction.

### Data processing

All data processing steps were performed using the *muon* (version 0.1.3)^82^ and *anndata* (version 0.8.0)^83^ Python packages.

#### TCGA pan-gynecologic atlas

All data modalities, survival data and metadata were downloaded using the R package *curatedTCGAdata* (version 1.26.0)^17,80^. All data modalities except the protein array data were log-transformed. All data was centered and scaled to unit variance. Before the analysis, we selected the top 2000 highly variable features of the RNA-seq and methylation data sets.

#### Pan-cancer DepMap

We analyzed a multi-omic cancer cell line data set with proteomic data from Gonçalves et al. ^18^ combined with multi-omic and CRISPR essentiality data^19,48^ of the same cell lines. Processed and transcript per million (TPM) normalized RNA-seq data^49^, mutation data^50^ and integrated CRISPR essentiality scores were obtained from https://cellmodelpassports.sanger.ac.uk/^84^. Processed methylation beta values^50^ were obtained from https://www.cancerrxgene.org. Processed and normalized proteomics abundances and drug response lnIC50 data^50^ was obtained via figshare from Gonçalves et al.^18^. We log-transformed, centered and scaled the RNA-seq TPM data and the pre-processed methylation beta values and selected the top 2000 highly variable genes prior to the analysis. The proteomics data were imputed with zero, and the top 2000 highly variable proteins were selected. Additionally, we regressed out the sample mean as suggested in Gonçalves et al.^18^. The drug response lnIC50 values were centered and scaled.

#### Single-cell multi-omics of the human cerebral cortex

The single-cell RNA-seq and ATAC-seq data was obtained from Zhu et al.^20^ and downloaded in the h5ad format from https://cellxgene.cziscience.com/. RNA-seq data was normalized to library size and log-transformed. We selected the top 2000 highly variable genes in both modalities. Both data sets were centered and scaled before analysis. Cell type labels were obtained from the h5ad objects.

### Downstream analysis

#### Association testing

Associations between factors and mutations were tested using two-sample *t*-tests.

To test for associations between the guided SOFA factors and essentiality scores, we used factor values as predictors in a linear regression model to explain essentiality scores and then evaluated the significance of the fitted regression coefficient. Briefly, Genes with highly negative CRISPR-Cas9 essentiality scores are deemed essential, as their loss significantly impairs cell viability. Scores near-zero indicate nonessential genes with minimal impact on viability, while positive scores suggest that gene loss may confer a survival advantage. Therefore, negative coefficients (effect sizes) indicate that higher factor values correspond to lower essentiality scores, implying a decrease in cell viability. All *p* values were adjusted for multiple testing using the Benjamini-Hochberg procedure^85^.

#### Survival analysis

The survival analysis was performed using the *lifelines* package (version 0.27.7)^86^. Assessment of whether a factor was predictive of overall survival was done by per factor univariate Cox’s proportional hazard models. Resulting *p* values were adjusted for multiple testing using the Benjamini-Hochberg procedure^85^.

#### Gene set overrepresentation analysis (ORA)

To annotate the factors with biological processes we used the enrichr API^87^ provided in the *gseapy* Python package (version 1.0.4)^88^. We used the top 100 features with highest or lowest loadings of a given factor and performed gene set overrepresentation analysis using all genes in the data set as background.

#### Fraction of explained variance

Assuming the data **X** is centered, the fraction of variance that is explained by factor *k* of modality *m* was calculated as follows:

3 $${R}^{2}=1-\frac{{\sum }_{i=1}^{N}{\sum }_{j=1}^{{D}^{m}}{({z}_{{ik}}\times {w}_{{kj}}^{m})}^{2}}{{\sum }_{i=1}^{N}{\sum }_{j=1}^{{D}^{m}}{{x}_{{ij}}}^{2}}$$

Where the denominator corresponds to the total sum of squares of the centered data Χ and the numerator to the residual sum of squares. The fraction can be understood as the variability a given factor *k* accounts for.

#### Two-dimensional visualizations of the latent space

The t-distributed Stochastic Neighbor Embeddings (tSNE)^89^ for 2D visualizations of the latent space were calculated using the *scikit-learn* Python package (version 1.2.1)^90^. Uniform Manifold Approximation and Projection (UMAP)^91^ embeddings were computed using the implementation provided in the *scanpy* Python package (version 1.9.3)^92^. We chose to visualize the latent space of the TCGA and DepMap data sets using tSNE and UMAP for the single-cell multiome data set. This choice was made for visualization clarity, as UMAP produced more interpretable plots for data sets with larger numbers of data points.

#### Root mean square error for model selection

To assess the quality of the individual model fits we calculated the root mean square error as follows:

4 $${{\rm{RMSE}}}=\frac{\sqrt{{\sum }_{m=1}^{M}{\sum }_{i=1}^{M}{\sum }_{j=1}^{D}{({x}_{{ij}}^{m}-{\hat{x}}_{{ij}}^{m})}^{2}\left.\right)}}{{\sum }_{m=1}^{M}N\times {D}_{m}}$$

#### Adjusted Rand index

To verify that the unguided factors did not capture variance associated with categorical guiding variables, such as cancer type labels, we compared the Adjusted Rand Index (ARI)^93^ for clusterings on the latent factors of a guided vs unguided model. To this end, we used the *scib* package^94^ to perform clustering on the latent factors and calculate the ARI between the cluster labels and categorical guiding variables for 10 independent initializations. The ARI measures how well the clusterings align with the guiding variables; a higher ARI corresponds to greater overlap, thus indicating that the latent factors captured variance associated with the guiding variables.

#### RV coefficient

The RV coefficient measures the similarity of two subspaces and can be seen as the multivariate analogue of the Pearson correlation coefficient. It is rotation-, permutation-, and sign-invariant and takes values between 0 and 1, where values closer to 1 indicate higher similarity^95^. It is calculated as:

5 $${RV}\left(A,B\right)=\frac{{tr}(A{A}^{T}B{B}^{T})}{\sqrt{{tr}[{(A{A}^{T})}^{2}]{tr}[{(B{B}^{T})}^{2}]}}$$

Where A and B correspond to the loadings of two SOFA models with different initializations.

### List of software used

For the data analysis presented in this paper the following open-source Python(3.8.5) packages were used: anndata (0.8.0), biopython(1.81), gseapy(1.0.4), matplotlib(3.7.3), mofapy(0.7.0), mofax(0.3.6), muon(0.1.3), numpy(1.23.5), pyro-ppl(1.7.0), scanpy(1.9.3), scikit-learn(1.2.1), scipy(1.10.1), seaborn(0.12.1), statsmodels(0.14.0).

### Reporting summary

Further information on research design is available in the Nature Portfolio Reporting Summary linked to this article.

## Supplementary information

Supplementary Information

Reporting Summary

Transparent Peer Review file

## Source data

Source Data

## Data Availability

Processed data to reproduce the analysis and figures are available on Zenodo 10.5281/zenodo.14761127.Source data are provided with this paper.

## Code availability

SOFA is implemented in the Python package *biosofa* and is available on github (https://github.com/tcapraz/SOFA) and PyPI (https://pypi.org/project/biosofa/), along with documentation and example analysis notebooks. Code and processed data to reproduce the analysis and figures are available on Zenodo 10.5281/zenodo.14761127. The package version used to perform the presented analyses and generate the figures are available on Zenodo https://zenodo.org/records/19594991.
