# Supplementary material for: Semi-supervised Omics Factor Analysis (SOFA) disentangles known and latent sources of variation in multi-omic data
Source: Nat Commun. 2026 Aug 20;17:8725. doi: 10.1038/s41467-026-74694-6 (PMC13493367; doi:10.1038/s41467-026-74694-6)
Supplement: Supplementary file 1 [file 41467_2026_74694_MOESM1_ESM.pdf]

Semi-supervised Omics Factor Analysis (SOFA) disentangles known and latent sources of variation in multi-omic data

Capraz et al., Nature Communications (2026)

Supplementary Methods

1 Setup

SOFA is based on group factor analysis (GFA), a multivariate statistical technique that describes linear relationships between groups of variables that were measured on a common set of objects or *samples*. Each group represents a set of particularly related variables [6]. Multi-Omics Factor Analysis (MOFA, Argelaguet et al. [1]) is an adaptation of this idea to multi-omics data in biology, where a series of biological specimen are assayed using multiple 'omics technologies, also called *views*. Each view corresponds to a group of variables. A typical objective in applications of these methods is to find a lower dimensional representation of the data that captures correlation structures that are shared across the views, as well as correlation structures among variables within, and unique to, individual views. Mathematically, these correlation structures are represented by factors (see below).

SOFA extends GFA by enabling the analyst to "guide" a subset of the factors by explicitly known guide variables for the samples, while others are assumed to be unobserved, or *latent*. The parameters of the model are inferred using black box variational inference [8].

We consider data $\{\mathbf{X}^1, \ldots, \mathbf{X}^M, \mathbf{Y}^1, \ldots, \mathbf{Y}^C\}$, where $\mathbf{X}^m \in \mathbb{R}^{N \times D^m}$ is the matrix of measurements for the m-th view, for N samples with D^m features each, and $\mathbf{Y}^c \in \mathbb{R}^N$ is the vector of measurements of the c-th known guide variable. The samples are the same, and aligned, across modalities and variables. There is no assumption of relationships or alignments between the features from the different views. SOFA finds a reduced-dimensional representation of these data using $K \leq \min\{D^1, \ldots, D^M\}$ factors:

$$\mathbf{X}^m = \mathbf{Z} \times \mathbf{W}^m + \boldsymbol{\varepsilon}^m, \qquad m = 1, \ldots, M \tag{1}$$

with the latent factors $\mathbf{Z} \in \mathbb{R}^{N \times K}$, the loadings $\mathbf{W^m} \in \mathbb{R}^{K \times D^m}$, and the residuals $\boldsymbol{\varepsilon}^m \in \mathbb{R}^{N \times D^m}$. We model the residuals as independent random variables from Normal distributions with feature- and view-specific standard deviation σ_j^m.

$$\varepsilon_{ij}^m \sim \mathcal{N}(0, \sigma_j^m) \qquad \forall \qquad i = 1, \ldots, N \qquad j = 1, \ldots, D^m. \tag{2}$$

This implies for the data

$$x_{ij}^m \sim \mathcal{N}\left(\sum_{k=1}^{K} z_{ik} w_{kj}^m \, , \sigma_j^m\right). \tag{3}$$

We set no priors on the standard deviation parameters σ_j^m.

For model inference, we first set aside the variables $\mathbf{Y}^c$ (we bring them back in Section 2). We posit standard Normal priors on the factors,

$$z_{ik} \sim \mathcal{N}(0, 1), \tag{4}$$

and hierarchical horseshoe priors [4] on the loadings $\mathbf{W^m}$. This assumption encourages sparsity, facilitates model interpretability, and is justified by the observation that many useful models of biological systems use sparse sets of relationships between the system components. To this end, we introduce the parameters λ_{kj}^m, δ_k^m and τ^m and assume half Cauchy priors for them:

$$\begin{aligned} \tau^m &\sim \mathcal{C}^+(0, 1) \\ \delta_k^m &\sim \mathcal{C}^+(0, 1) \\ \lambda_{kj}^m &\sim \mathcal{C}^+(0, 1). \end{aligned} \tag{5}$$

The parameter τ^m is used to generate view-level sparsity (i.e., whole views can be deactivated), δ_k^m generates factor-level sparsity (i.e., whole factors can be deactivated within individual views), and λ_{kj}^m generates feature-level sparsity (i.e., individual features can be deactivated within individual factors and views). Then,

$$w_{kj}^m \sim \mathcal{N}(0, \lambda_{kj}^m \delta_k^m \tau^m). \tag{6}$$

2 Adding factor guidance

SOFA associates the first C factors with the guide variables $\mathbf{Y}^1, \ldots, \mathbf{Y}^C$ by linear regression. Intuitively, this constrains this set of "guided" factors to explain sources of variation in $\mathbf{X}^1, \ldots, \mathbf{X}^M$ with factors that are highly correlated with the variables $\mathbf{Y}^1, \ldots, \mathbf{Y}^C$. Conversely, the remaining unguided factors are free to capture further sources of variation in $\mathbf{X}^1, \ldots, \mathbf{X}^M$ that are less correlated with the guide variables.

Thus, we posit a generalized linear model [7]

$$f\left(\mathbb{E}[y_i^c]\right) = \alpha_c + \beta_c z_{ic}, \tag{7}$$

where f is a link function, and we assume a standard Normal prior on the offset and slope coefficients,

$$\alpha_c, \beta_c \sim \mathcal{N}(0, 1). \tag{8}$$

SOFA supports continuous and categorical response variables by supporting Normal, Bernoulli and Categorical likelihood functions. For real-valued continuous variables, link function and likelihood are

$$f(y) = y \tag{9}$$

$$y_i^c \sim \mathcal{N}\left(\alpha_c + \beta_c z_{ic}, \kappa_c^2\right) \tag{10}$$

where κ_c^2 denotes the noise variance. For binary variables,

$$f(p) = \log \frac{p}{1-p} \tag{11}$$

$$y_i^c \sim \text{Bernoulli}\left(f^{-1}(\alpha_c + \beta_c z_{ic})\right). \tag{12}$$

For a categorical variable with L levels,

$$f^{-1}(x_1, \ldots, x_L) = \frac{1}{\sum_{l'=1}^{L} e^{x_{l'}}} (e^{x_1}, \ldots, e^{x_L}) \tag{13}$$

$$y_i^c \sim \text{Categorical}\left(f^{-1}(\boldsymbol{\alpha}_c + \boldsymbol{\beta}_c z_{ic})\right), \tag{14}$$

where $\boldsymbol{\alpha}_c, \boldsymbol{\beta}_c$ are vectors of length L whose elements come from the prior (8).

3 Inference

For the implementation of SOFA, we used the probabilistic programming framework Pyro [2]. In the following, we describe the inference for the case that all $\mathbf{Y}_c$ are normal, as in Eqn. (10). The likelihood is then

$$\begin{aligned} p(\mathbf{X}, \mathbf{Y}, \mathbf{Z}, \mathbf{W}, \boldsymbol{\lambda}, \boldsymbol{\beta}, \boldsymbol{\tau}, \boldsymbol{\delta}) = \\ \prod_{m=1}^{M} \prod_{i=1}^{N} \prod_{j=1}^{D^m} \mathcal{N}(x_{ij}^m \mid \sum_{k=1}^{K} z_{ik} w_{kj}^m \lambda_{kj}^m \delta_k^m \tau^m, \sigma_j^m) \\ \prod_{i=1}^{N} \prod_{c=1}^{C} \mathcal{N}(y_i^c \mid \alpha_c + z_{ic}\beta_c, \kappa_c^2) \\ \prod_{i=1}^{N} \prod_{k=1}^{K} \mathcal{N}(z_{ik} \mid 0, 1) \\ \prod_{m=1}^{M} \prod_{j=1}^{D^m} \prod_{k=1}^{K} \mathcal{N}(w_{kj}^m \mid 0, 1)\, \mathcal{C}^+(\lambda_{kj}^m \mid 0, 1)\, \mathcal{C}^+(\delta_k^m \mid 0, 1)\, \mathcal{C}^+(\tau^m \mid 0, 1) \\ \prod_{c=1}^{C} \mathcal{N}(\beta_c \mid 0, 1) \end{aligned} \tag{15}$$

We make use of the mean field assumption [3] and introduce a factorized family of parameterized distributions to approximate the intractable posterior,

$$q_\phi(\Theta) = \prod_{\theta \in \Theta} q_\phi(\theta), \tag{16}$$

where Θ denotes the collection of inferred parameters $\{\mathbf{Z}, \mathbf{W}, \boldsymbol{\lambda}, \boldsymbol{\beta}, \boldsymbol{\tau}, \boldsymbol{\delta}\}$ and ϕ the parameters of the variational distribution $q(\cdot)$. For the variational distribu-

tion, we posit the following form using Normal and log-Normal distributions:

$$
\begin{aligned}
q(\mathbf{Z}, \mathbf{W}, \boldsymbol{\lambda}, \boldsymbol{\beta}, \boldsymbol{\tau}, \boldsymbol{\delta}) = &\prod_{i=1}^{N} \prod_{k=1}^{K} \mathcal{N}(z_{ik} \mid m_{ik}, s_{ik}) \\
&\prod_{m=1}^{M} \prod_{j=1}^{D^m} \prod_{k=1}^{K} \mathcal{N}(w_{kj}^m \mid m_{kj}^m, s_{kj}^m) \\
&\prod_{m=1}^{M} \prod_{j=1}^{D^m} \prod_{k=1}^{K} \log\mathcal{N}(\lambda_{kj}^m \mid m_{kj}^m, s_{kj}^m) \\
&\prod_{m=1}^{M} \prod_{k=1}^{K} \log\mathcal{N}(\delta_k^m \mid m_k^m, s_k^m) \\
&\prod_{m=1}^{M} \log\mathcal{N}(\tau^m \mid m^m, s^m) \\
&\prod_{c=1}^{C} \mathcal{N}(\beta_c \mid m_c, s_c)
\end{aligned}
\tag{17}
$$

We optimize the evidence lower bound (ELBO) with respect to the variational parameters $\phi = \{\mathbf{m}, \mathbf{s}\}$. $\mathbf{m}$ and $\mathbf{s}$ are the means and standard deviations of the factors of the variational distribution in Eqn. (17), where we dropped the indices for notational simplicity. The ELBO is given by:

$$
\text{ELBO}(\phi) = \mathbb{E}_{q_\phi}[\log p(\mathbf{X}, \mathbf{Y}, \boldsymbol{\Theta}) - \log q_\phi(\boldsymbol{\Theta})]. \tag{18}
$$

The ELBO is optimized using stochastic variational inference by taking Monte Carlo samples from the variational distribution and computing unbiased but noisy gradients with respect to the variational parameters from the ELBO [5, 8]. The noise scales σ for $\mathbf{X}$ and κ^2 for $\mathbf{Y}$ are fit via maximum likelihood, and no prior distributions are imposed on them. Optimization is stopped when the change in ELBO is smaller than a user defined threshold over the last 2000 iterations.

If necessary for large datasets, e.g., in applications to single-cell RNA-sequencing data, we compute the gradients over subsamples of batches of the observations.

SUPPLEMENTARY FIGURES

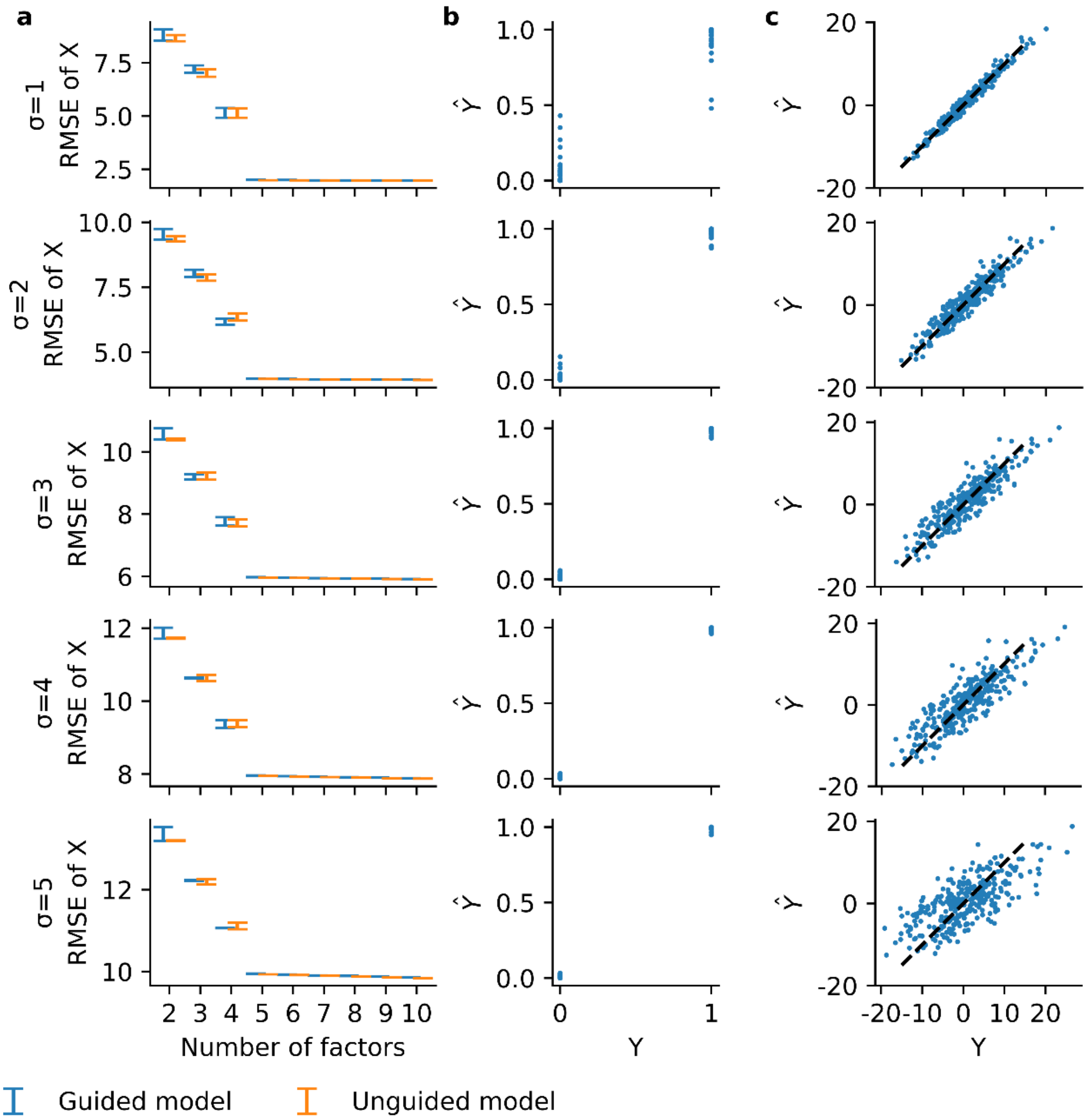

Supplementary Figure S1: RMSE of guided and unguided SOFA models for simulations with different noise levels. In-silico data were simulated according to the generative model of SOFA using a true rank of 5 and two guiding variables for the first (binary) and second factor (continuous). The simulated multi omic data comprised 2 views with each 300 observations and 2000 features. To assess the model fit and selection, we varied the noise variance σ (rows) and performed the SOFA factorization over a range of decomposition ranks with and without the use of guiding variables. a: Average root mean square error (RMSE) of true and predicted data averaged over 10 model initializations. Error bars indicate mean values +/- the standard deviation. **b-c**: Scatter plot of true guiding variable (Y) vs predicted guiding variable (Ŷ) (**b**: binary; **c**: continuous) of one of the rank-5 models from **a**.

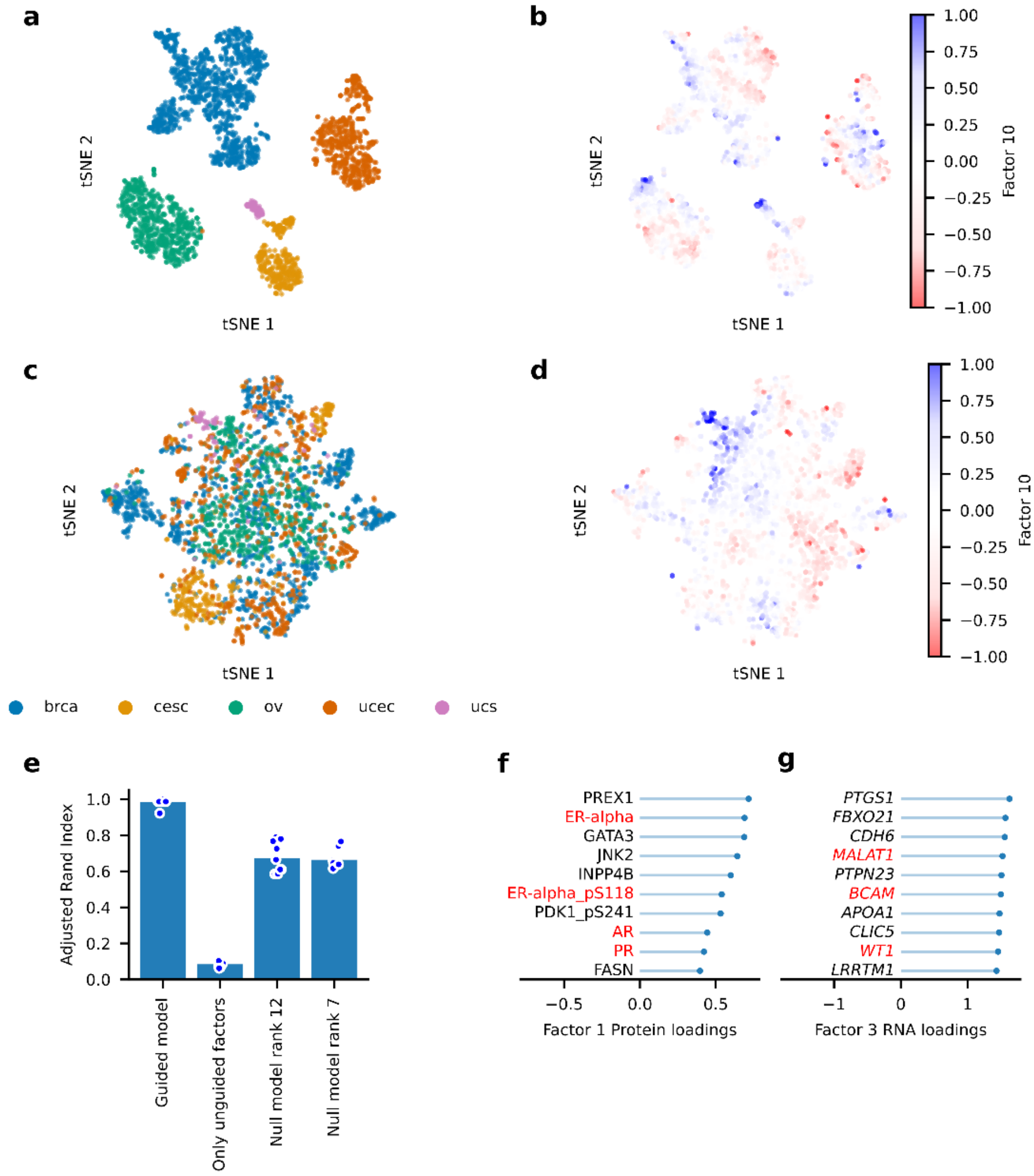

Figure S2: Comparison of latent spaces captured by guided and unguided factors in the TCGA[9] data set. a: tSNE of all factors colored by cancer type. **b**: tSNE of all factors colored by Factor 10. **c**: tSNE of unguided factors 7-12 colored by cancer type. **d**: tSNE of unguided factors 7-12 colored by Factor 10. **e**: Adjusted Rand Index (ARI) for clusterings of all factors of the guided model, only unguided Factors 7-12, unguided null model of rank 12 and unguided null model of rank 7. The clusterings were compared to the true cancer type labels. A higher ARI suggests better overlap of the clustering with the cancer type labels. The ARI was computed for 10 independent model initializations. Bars indicate mean values +/- the standard deviation as error bars. **f**: Highest protein loadings of Factor 1. **g**: Highest RNA loadings of Factor 3.

brca: breast invasive carcinoma; cesc: Cervical squamous cell carcinoma and endocervical adenocarcinoma; ov: Ovarian serous

cystadenocarcinoma; ucec: Uterine Corpus Endometrial Carcinoma; ucs: Uterine Carcinosarcoma

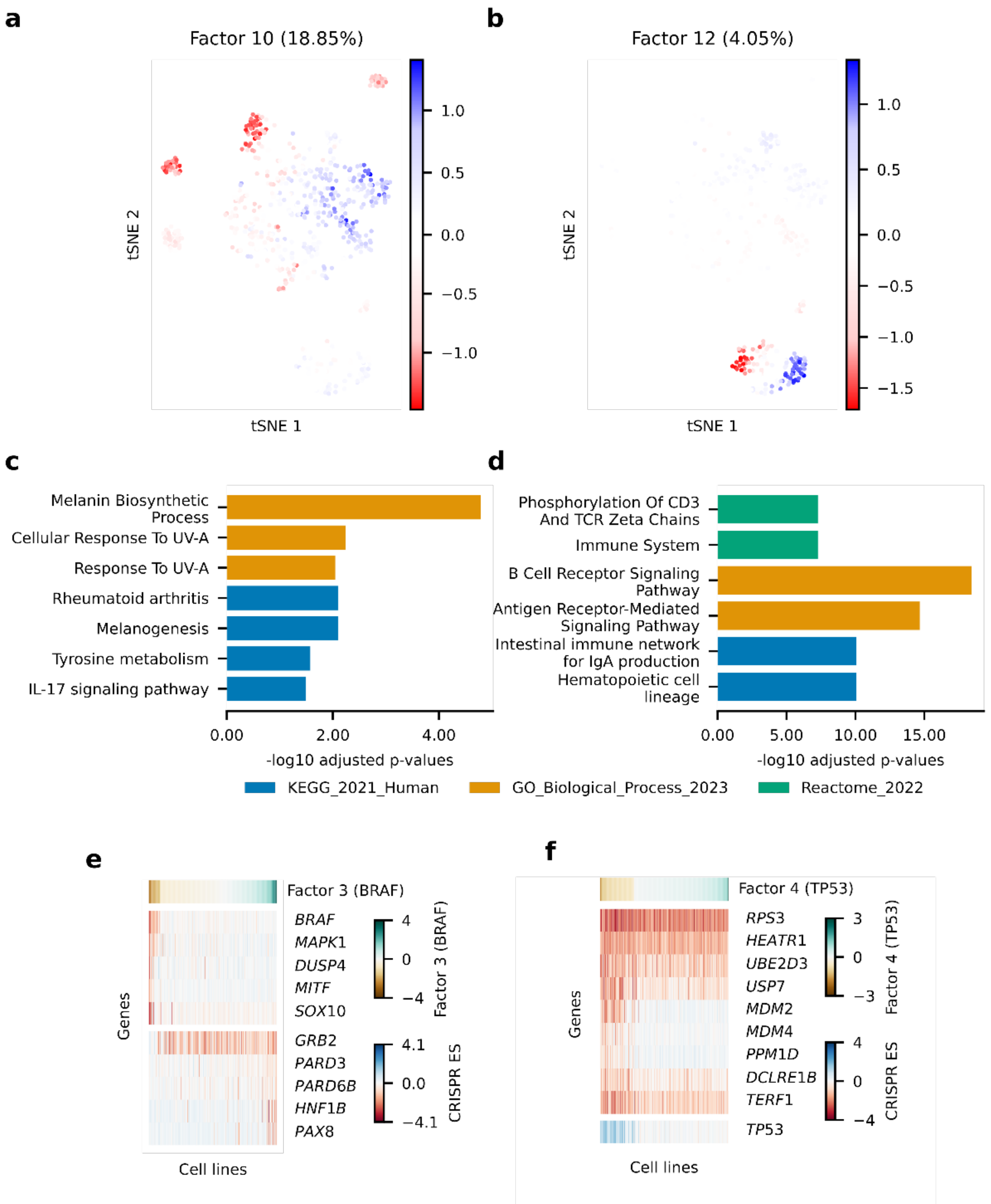

Figure S3: Gene set overrepresentation analysis and CRISPR essentiality features associated with guided factors in the DepMap[10-12] data set. a-b: tSNE of all factors coloured by the values of factors 10 and 12. The fraction of explained variance is shown in parenthesis. **c**: Gene set overrepresentation analysis (one-sided hypergeometric test) of highest RNA loadings of factor 3 (BRAF mutation). **d**: Gene set overrepresentation analysis (one-sided hypergeometric test) of highest RNA loadings of factor 6 (hematopoietic lineage). **e**: Heatmap showing CRISPR essentiality scores (ES) of top 10 genes significantly associated with Factor 3 (BRAF mutation), tested using a linear model (two-sided t-test of the slope coefficient; n=778). The cell lines are ordered based on their values of Factor 3. **f**: Heatmap showing CRISPR essentiality scores (ES) of top 10 genes significantly associated with Factor 4 (TP53

mutation), tested using a linear model (two-sided t-test of the slope coefficient; n=778). The cell lines are ordered based on their values of Factor 4.

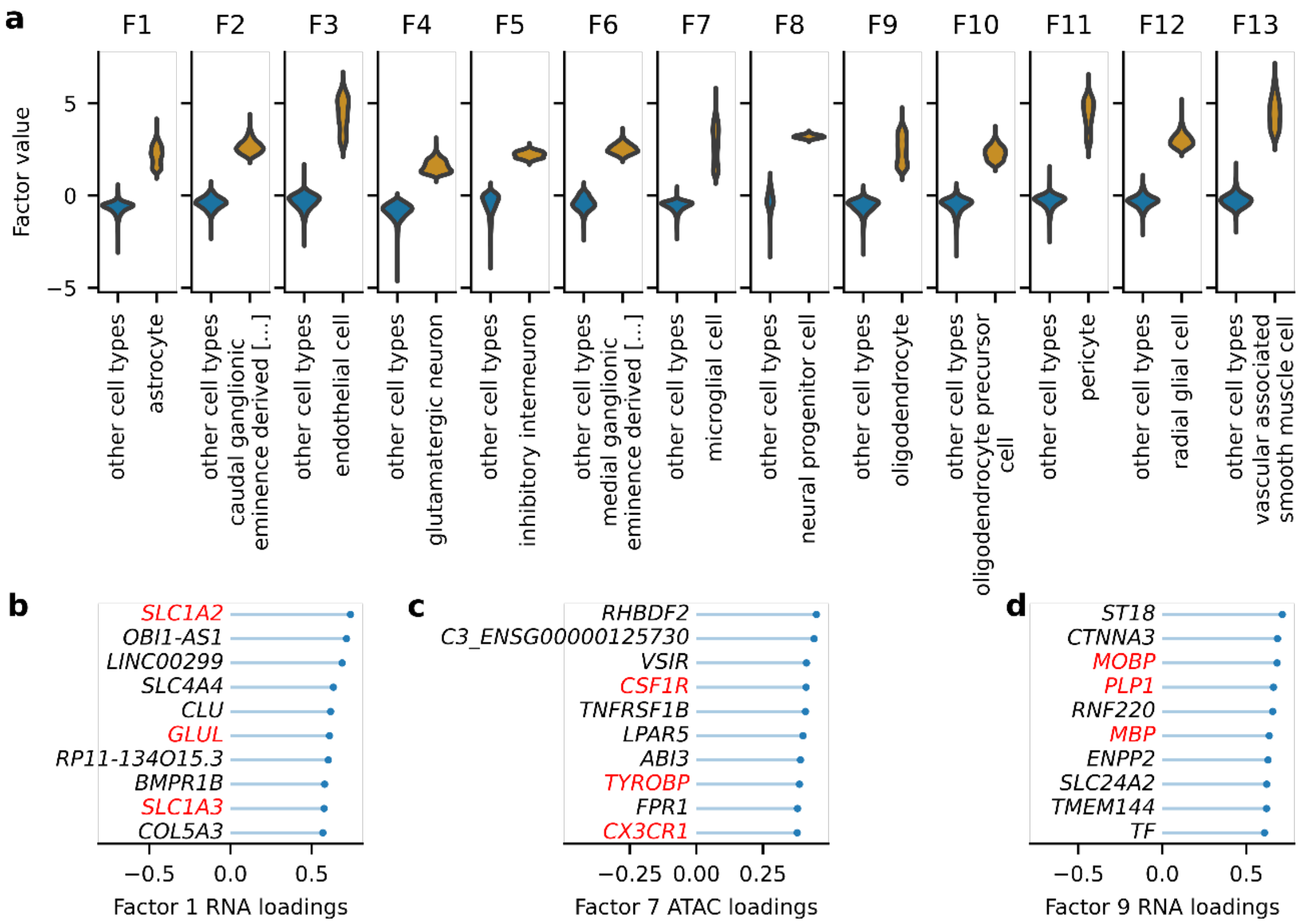

Figure S4: Guided factors are specific to cell type labels and capture cell type markers in single-cell multiome data[13]. a: Violin plots of each guided factor vs their guiding cell type label. **b**: Highest transcriptomics (RNA) loadings of factor 1 (astrocytes). **c**: Highest ATAC loadings of factor 7 (microglial cells). **d**: Highest transcriptomics (RNA) loadings of factor 9 (oligodendrocytes).

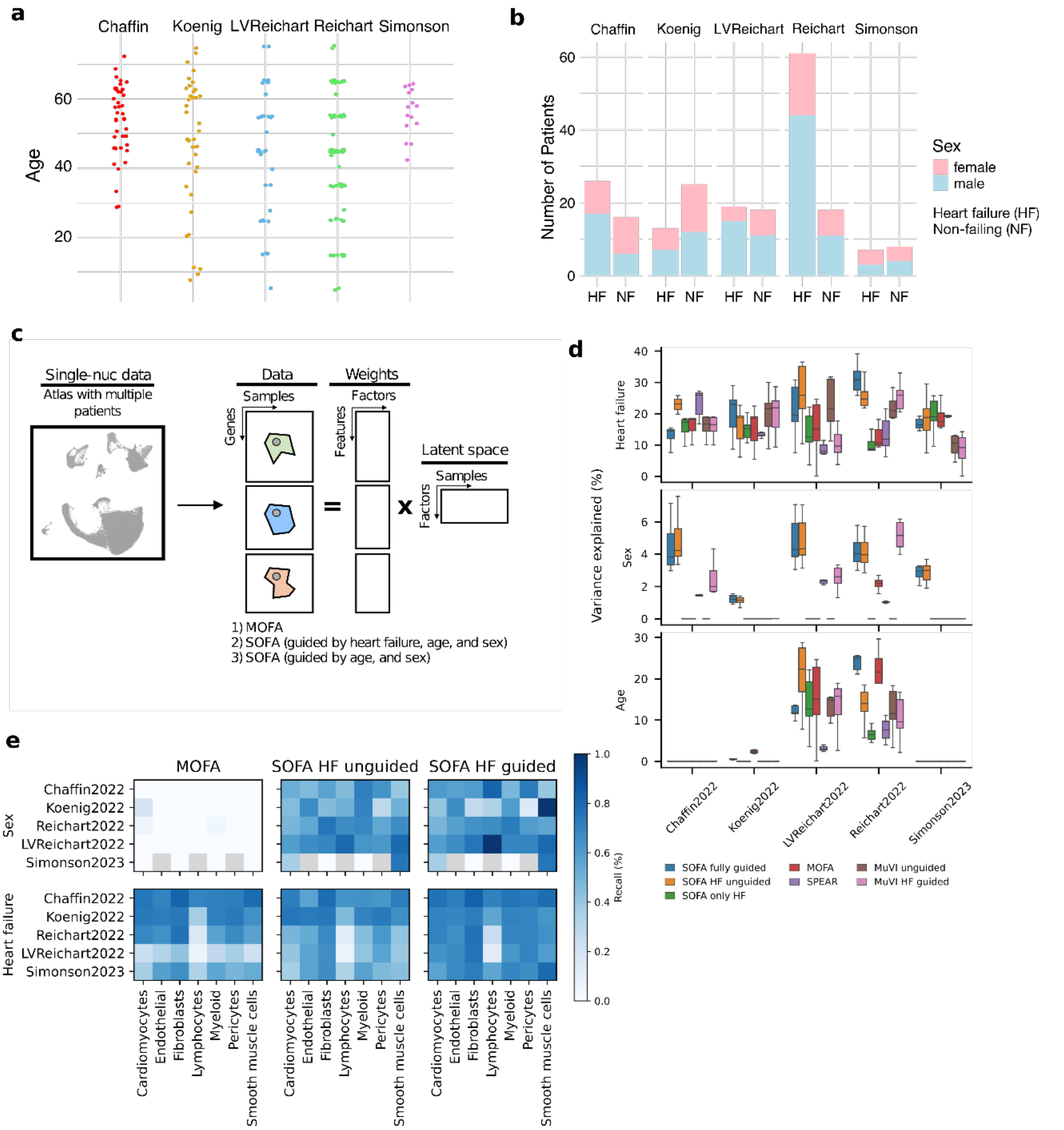

Figure S5: Comparison between SOFA and state-of-the-art Factor models in the inference of multicellular programs from single-nuclei RNA-seq data[14-19]. **a**: Age distribution across five patient cohorts. **b**: Sex distribution across five patient cohorts grouped by heart failure condition. **c**: Schematic of application of SOFA and MOFA for multicellular integration. Single-nuclei data is transformed into a multiview representation, where each view is the pseudobulk gene expression matrix of a cell-type. The latent space represents coordinated gene expression programs across cell-types or multicellular programs. **d**: Box plots with fraction of variance explained associated with heart failure, sex, and age across distinct data sets and models. (center line: median, box limits: 25th percentile and 75th percentile, whiskers: 1.5 interquartile range) **e**: Recall of differentially expressed genes for sex and heart failure conditions,

based on gene sampling of equivalent size from the loading scores of the factors most strongly associated with each condition.

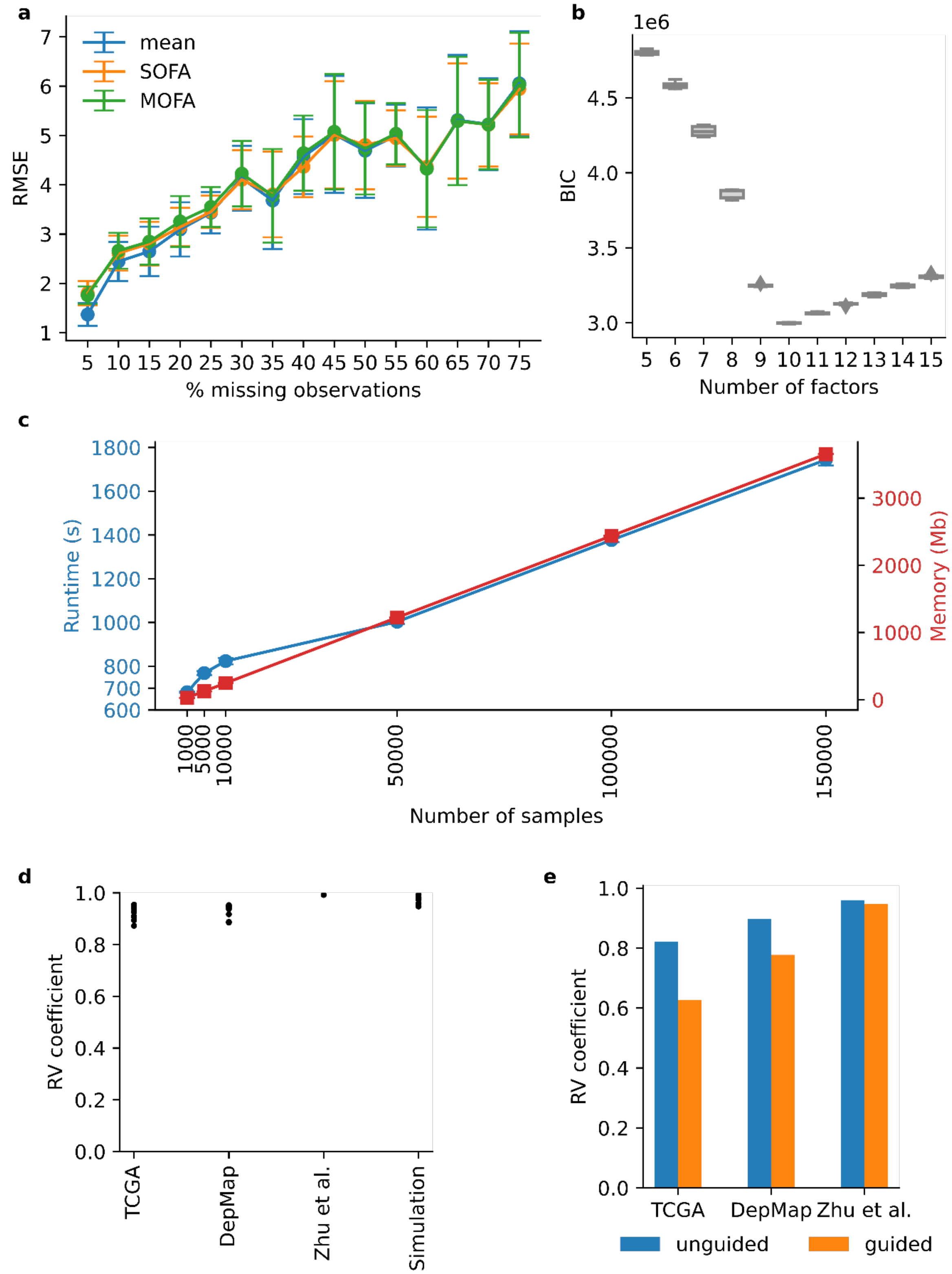

Figure S6: Scalability, computational resource requirements and robustness of SOFA to multiple different initializations and missing values. a: RMSE of SOFA, MOFA and mean imputation of 10 simulations with increasing percentage of missing values. Simulated data consisted of three views, with 2000 features each and 200 samples. Error bars indicate mean values +/- the standard deviation. **b**: BIC across

10multiple SOFA fits for simulated data with true rank 10. (center line: median, box limits: 25th percentile and 75th percentile, whiskers: 1.5 interquartile range) **c**: Runtime (seconds) and memory (Mb) requirements for 10 independent simulations with three views and two guiding variables and increasing number of samples. **d**: RV coefficients between 10 independent initializations of SOFA for the TCGA[9], DepMap[10-12], scMultiome[13] and a simulation with three views, two guiding variables and 200 samples. **e**: RV coefficients between a MOFA model and unguided (blue) and guided (orange) SOFA model for the TCGA[9], DepMap[10-12] and scMultiome[13] data sets.

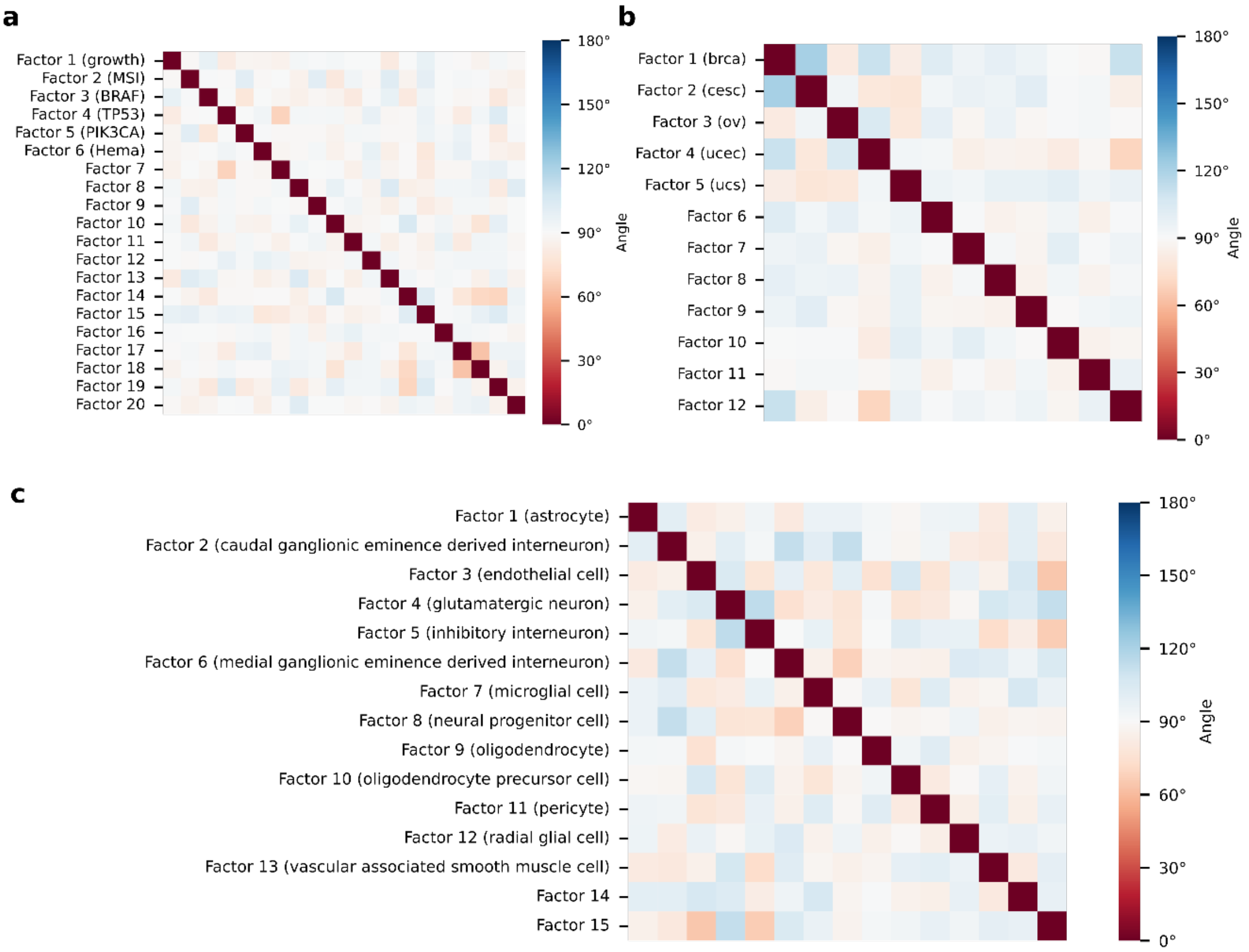

Figure S7: Angles between factors for the different data sets. a: TCGA[9], **b:** DepMap[10-12], **c**: scMultiome[13].

REFERENCES

1. Argelaguet, R. et al. Multi-omics factor analysis—a framework for unsupervised integration of multi-omics data sets. Molecular systems biology, 14(6):e8124. (2018).

2. Bingham, E. et al. Pyro: Deep universal probabilistic programming. Journal of machine learning research, 20(28):1–6. (2019).

3. Blei, D. M., Kucukelbir, A., and McAuliffe, J. D. Variational inference: A review for statisticians. Journal of the American statistical Association, 112(518):859–877. (2017).

4. Carvalho, C. M., Polson, N. G., and Scott, J. G. Handling sparsity via the horseshoe. In Artificial intelligence and statistics, pages 73–80. PMLR. (2009).

5. Hoffman, M. D., Blei, D. M., Wang, C., and Paisley, J. Stochastic variational inference. Journal of Machine Learning Research. (2013).

6. Klami, A., Virtanen, S., Leppäaho, E., and Kaski, S. Group factor analysis. IEEE transactions on neural networks and learning systems, 26(9):2136–2147. (2014).

7. Nelder, J. A. and Wedderburn, R. W. Generalized linear models. Journal of the Royal Statistical Society Series A: Statistics in Society, 135(3):370–384. (1972).

8. Ranganath, R., Gerrish, S., and Blei, D. Black box variational Inference. In Artificial intelligence and statistics, pages 814–822. PMLR. (2014).

9. Cancer Genome Atlas Research Network et al. The Cancer Genome Atlas Pan-Cancer analysis project. Nat. Genet. 45, 1113–1120 (2013).

10. Gonçalves, E. et al. Pan-cancer proteomic map of 949 human cell lines. Cancer Cell 40, 835–849.e8 (2022).

11. Boehm, J. S. et al. Cancer research needs a better map. Nature 589, 514–516 (2021).

12. Pacini, C. et al. Integrated cross-study datasets of genetic dependencies in cancer. Nat. Commun. 12, 1661 (2021).

13. Zhu, K. et al. Multi-omic profiling of the developing human cerebral cortex at the single-cell level. Sci Adv 9, eadg3754 (2023).

14. Ramirez Flores, R. O., Lanzer, J. D., Dimitrov, D., Velten, B. & Saez-Rodriguez, J. Multicellular factor analysis of single-cell data for a tissue-centric understanding of disease. Elife 12, (2023).

15. Lanzer, J. D., Ramirez Flores, R. O., Liñares Blanco, J. & Saez-Rodriguez, J. A cross-study transcriptional patient map of heart failure defines conserved multicellular coordination in cardiac remodeling. bioRxiv (2024) doi:10.1101/2024.11.04.621815.

16. Chaffin, M. et al. Single-nucleus profiling of human dilated and hypertrophic cardiomyopathy. Nature 608, 174–180 (2022).

17. Koenig, A. L. et al. Single-cell transcriptomics reveals cell-type-specific diversification in human heart failure. Nat. Cardiovasc. Res. 1, 263–280 (2022).

18. Reichart, D. et al. Pathogenic variants damage cell composition and single cell transcription in cardiomyopathies. Science 377, eabo1984 (2022).

19. Simonson, B. et al. Single-nucleus RNA sequencing in ischemic cardiomyopathy reveals common transcriptional profile underlying end-stage heart failure. Cell Rep. 42, 112086 (2023).
