# Supplementary material for: Semi-supervised Omics Factor Analysis (SOFA) disentangles known and latent sources of variation in multi-omic data
Source: Nat Commun. 2026 Aug 20;17:8725. doi: 10.1038/s41467-026-74694-6 (PMC13493367; doi:10.1038/s41467-026-74694-6)
Supplement: Supplementary file 3 [file 41467_2026_74694_MOESM3_ESM.pdf]

nature portfolio

Peer Review File

Semi-supervised Omics Factor Analysis (SOFA) disentangles known and latent sources of variation in multi-omic data

Corresponding Author: Dr Tümay Capraz

This file contains all reviewer reports in order by version, followed by all author rebuttals in order by version.

Version 0:

Reviewer comments:

Reviewer #1

(Remarks to the Author)
The manuscript entitled "Semi-supervised Omics Factor Analysis (SOFA) disentangles known and latent sources of variation in multi-omics data" proposes very interesting problem in multi-omics latent factor analysis. Given the increasing availability of multi-omics datasets out of several disease studies, it is extremely important to develop new statistical and analytical methods exploring the hidden structure and variation in such datasets to uncover and understand the novel biology. Most of the previous studies carried out unsupervised studies, and a few supervised studies, to identify the latent structure. SOFA proposes a semi-supervised method that utilizes known factors as guiding variables (e.g. disease subtype, gender, age, batch effects etc) to identify unknown hidden structure (latent factors) in the data. The main idea of the method proposed here is to regress out the known drivers of technical or known biological sources of variation from the latent space so that remaining hidden factors can be better identified. The method builds upon previously proposed Multi-Omics Factor Analysis (MOFA) and generalized regression with known covariates which the authors call as guiding variables.

Overall, the method proposed in the manuscript is very interesting, very well written, and is likely to contribute to the literature. The code is well written and well documented, and likely to be useful for users with a strong statistical background with expertise in Python. However, I feel that a few more clarification and elaboration is needed before publication.

Below are some of my comments:

• The guiding variables (covariates) are modeled as a function of guided variables factors) in the model. It is not clear if the authors consider one guiding variable and one guided vector per function e.g. g1, g2 etc (Fig1b and methods section), or if more than one guided variable can be modelled within those functions. It is common to have fixed and only a few guiding variables in most cases. However, guided factors can be one or more depending on the data. I recommend authors to clarify that in the manuscript.
• How is the optimum number of factors determined? The identification of the number of factors with justification should be provided/elaborated in the methods section.
• While incorporation of the known a few covariates in the SOFA method performs reasonably well in the pattern recognition, adjustment of too many covariates may shrink true hidden pattern of interest and the hidden signal may not be identifiable in the residual data. This could be the limitation of the approach.
• Authors have presented comparison of the proposed SOFA with existing MOFA methods using heart failure single cell data. I wonder how the comparisons would come out if they had compared the methods in other three examples too.

(Remarks on code availability)
The code looks good. The code is written in Python and is usable for those who knows Python. It would have been more helpful for broader community if the code had been written in R. However, this is my personal opinion and has nothing to do with my review.

Reviewer #2

(Remarks to the Author)
The manuscript introduces Semi-supervised Omics Factor Analysis (SOFA), a novel computational method for analyzing

multi-omic datasets by integrating known covariates into a factor analysis framework. SOFA partitions latent factors into guided components (linked to specified variables) and unguided components (capturing novel variation), aiming to disentangle known and unexpected patterns in biological data. The method is demonstrated across diverse datasets, including cancer (TCGA, DepMap), single-cell brain development, and heart failure, showcasing its ability to uncover biologically relevant insights. Strengths include its innovative semi-supervised approach, robust Bayesian implementation, and broad applicability. However, several areas require enhancement to strengthen its contribution prior to publication.

Comments:
(1) The manuscript compares SOFA to Multi-Omics Factor Analysis (MOFA) in the heart failure analysis (lines 314-359) and briefly references methods like Group Factor Analysis (GFA), Principal Component Analysis (PCA), Partial Least Squares (PLS), SPEAR, and DIABLO (lines 57-85). However, it lacks a comprehensive comparison with other semi-supervised or supervised multi-omics tools, such as iCluster or MuVI. While quantitative metrics (e.g., variance explained, lines 339-341, Figure S5d) are provided, a detailed discussion of how SOFA's guided factor strategy outperforms or complements these methods would better articulate its unique contribution. For instance, how does SOFA address specific limitations of MOFA's unsupervised nature or DIABLO's emphasis on known covariates?
(2) SOFA relies on user-specified guiding variables (e.g., cancer types, cell labels) to structure its factors (lines 103-116), which may limit its utility when covariates are unclear or unavailable. The authors should acknowledge this constraint and consider suggesting strategies to identify potential guiding variables—such as unsupervised clustering or data-driven covariate selection—to enhance the method's flexibility.
(3) The use of stochastic variational inference supports scalability (lines 117-123), but the manuscript does not evaluate SOFA's performance with noisy or incomplete multi-omic data (e.g., missing modalities or technical artifacts). Including a robustness analysis under simulated noise conditions, building on the existing simulation framework (lines 466-478, Figure S1a), or discussing the impact of data quality on factor accuracy would bolster its practical credibility.
(4) SOFA outperforms MOFA in detecting low-variance signals, such as sex (lines 339-341, Figure S5d), attributed to its guided framework. However, the manuscript does not fully explore why MOFA underperforms beyond its unsupervised design. A deeper analysis of how SOFA's hierarchical Bayesian model or sparsity-inducing priors (lines 117-123) contribute to this advantage would clarify its technical superiority.
(5) The adoption of a hierarchical horseshoe prior and variational inference is central to SOFA (lines 117-123, Figure 1a), yet the manuscript justifies these choices primarily for scalability and sparsity without comparing them to alternatives (e.g., Gaussian priors, Markov Chain Monte Carlo [MCMC]). Providing a theoretical or empirical rationale—such as their specific role in enhancing guided versus unguided factor separation—would reinforce the method's design.
(6) While SOFA's implementation in Pyro is highlighted (lines 400-403), details on computational demands—such as runtime, memory usage, or scalability limits—are omitted. Including this information would enable users to assess its feasibility for large datasets, particularly in single-cell contexts with extensive sample sizes (e.g., lines 265-302, Figure 4).
(7) The technical definitions of guided and unguided factors are well-explained (lines 433-438), but their biological implications could be more accessible. For example, providing intuitive interpretations of findings like Factor 10 in TCGA (lines 167-189, Figures 2c-e)—such as "high values reflect increased cell proliferation, low values indicate immune activity"—would broaden the manuscript's appeal to non-specialists.

(Remarks on code availability)
The code is provided as an open-source Python package on GitHub and PyPI, with documentation and examples, facilitating reproducibility and adoption. Enhancing the documentation with tutorials that interpret typical outputs (e.g., factor distributions in Figure 2a or loading significance in Figure 3e) would further assist users in applying and understanding SOFA effectively.

Reviewer #3

(Remarks to the Author)
The authors propose SOFA, a semi-supervised probabilistic factor model designed to integrate multi-modal omics datasets, combining unsupervised latent structure discovery with guided supervision based on known clinical or biological covariates. The authors then proposed multiple experiments to show the effectiveness of the proposed SOFA on multiple applications and scenarios. SOFA is benchmarked against MOFA and demonstrates improved performance, particularly in scenarios involving small sample sizes and weak biological signals. This paper is clearly written and easy to understand. All figures are well demonstrated and with suitable annotations.

Comments:
1. SOFA only considers first-order features, ignoring potential two/higher order features. The authors implicitly assume that different guided features are independent, thus the guidance of factors to covariates is modeled via simple linear regression and there is no explicit modeling of nonlinearities, higher-order interactions. This assumption may not always hold in complex biological systems, which suggests that the unguided factors recalled by SOFA might be higher-order combinations of guided factors. The authors propose a simple experiment comparing the correlation between unguided factors with two/higher order combinations of guided factors.
2. No orthogonality constraint is imposed between guided and unguided factors, unguided factors may inadvertently absorb residual components of guided variables, potentially confounding biological interpretation.
3. The authors utilized a hierarchical horseshoe prior to shrink the loading weights on the view, which naturally makes me wonder about the performance of utilizing L1-normalization (LASSO) instead of horseshoe prior. The authors should perform further experiments validating this initialization method.
4. The model selection strategy is not described enough, as the selection of the number of factors is indeed the most

important hyperparameter in SOFA. This manuscript does not provide us a reproducible or standardized procedure for model selection, nor how these parameters used in these analyses are chosen, which may greatly limit the usage of proposed SOFA.
5. Not all unguided factors identified by SOFA are clearly explained or linked to known biological processes. For example, in the TCGA analysis, Factor 9 shows a pattern very similar to Factor 10, yet it is not discussed in the paper. Overall, the biological relevance of many unguided factors remains unclear. Are these factors capturing novel biological signals, or are they just modeling noise? It would be important for the authors to clarify how they distinguish meaningful factors from noise.
6. The scalability of SOFA on large-scale datasets was not reported in this paper.

(Remarks on code availability)
The code and documentation seem straightforward to follow.

Reviewer #4

(Remarks to the Author)
I co-reviewed this manuscript with one of the reviewers who provided the listed reports. This is part of the Nature Communications initiative to facilitate training in peer review and to provide appropriate recognition for Early Career Researchers who co-review manuscripts.

In this study, Capraz et al. introduce Semi-supervised Omics Factor Analysis (SOFA), a hierarchical Bayesian factor model designed to disentangle known covariates from novel axes of variation in multi-omic data. This work represents a substantial improvement over existing unsupervised approaches like MOFA by explicitly modeling sample metadata to distinguish biologically meaningful (surprising) factors from expected covariate-driven factors.

Major feedback:

- The manuscript clearly demonstrates SOFA's advantages over MOFA, addressing well-known challenges such as interpretation of latent factors. However, a more direct comparison with supervised matrix decomposition methods (DIABLO, SPEAR) would strengthen the argument for SOFA's unique advantages. Providing empirical evidence or a clear discussion contrasting these supervised approaches would further clarify the distinct contributions of SOFA.

- The manuscript lacks detailed discussion about critical methodological decisions. Clarifying the choice and determination process for the number of latent factors, as well as elaborating on practical computational considerations (e.g., runtime, memory requirements), would significantly enhance reproducibility and practicality.

- The authors briefly mention scalability ensured through stochastic variational inference but omit benchmarks or performance metrics. Including these details would aid readers in assessing SOFA's applicability to large and high-resolution datasets.

- The paper would benefit from explicitly addressing the robustness of variational inference, including information on multiple initializations and convergence diagnostics. This detail is crucial to instill confidence in the reproducibility of the inferred factors.

- A clearer explanation of the statistical assumptions (e.g., Gaussian likelihood assumptions and linear relationships between guided factors and covariates) would improve methodological transparency. Clarifying if SOFA can accommodate non-Gaussian modalities or nonlinear relationships would enhance its perceived flexibility.

- Critical methodological details mentioned in the online documentation, such as the adjustment of learning rates and the scaling factor for supervision strength, should also be explicitly described in the methods section of the manuscript.

Minor feedback:

- The manuscript exhibits inconsistencies in spelling (US vs. UK English); uniformity would enhance readability.

- The rationale behind choosing visualization methods (tSNE vs. UMAP) for figures 3 and 4 is not clearly stated. Clarifying these choices would improve the manuscript's coherence.

- SOFA's specification of Python=3.8 raises concerns due to its end-of-life status. Clarifying compatibility with current Python versions would support broader adoption.

- While the manuscript effectively validates guided and unguided factors through biological interpretation, additional external or prospective validation would strengthen claims of biological novelty. Discussing the potential or strategy for validating unguided factors using independent datasets could address this.

- The authors should acknowledge limitations such as potential complexity in selecting guiding variables and interpreting guided factors, as well as explicitly discuss the implications of the linear modeling assumption for covariates.

In summary, Capraz et al. present an innovative and valuable methodological contribution to multi-omics factor analysis. Addressing these additional methodological, comparative, and interpretational considerations will further strengthen the manuscript's clarity, impact, and utility to the community.

(Remarks on code availability)
The documentation of the code is sufficient. Some overall questions regarding code are also included in the comments to the authors.

Version 1:

Reviewer comments:

Reviewer #1

(Remarks to the Author)

(Remarks on code availability)
Code looks good.

Reviewer #3

(Remarks to the Author)
I have carefully reviewed the authors' rebuttal and the revised manuscript in response to the previous round of comments. I am satisfied with the authors' thorough and thoughtful responses to my concerns. The revisions have meaningfully improved the clarity, rigor, and overall presentation of the work. The authors have convincingly addressed my major concerns on the conceptual distinction between guided and unguided factors, biological interpretability and scalability. In particular, the methodological clarifications and additional analyses strengthen the validity of the proposed framework and its biological interpretations.
In its current form, the manuscript is substantially improved and I believe it now meets the standards for publication in Nature Communications. I therefore support acceptance of this work without further mandatory revisions.

(Remarks on code availability)

Reviewer #4

(Remarks to the Author)
All my comments have been sufficiently answered.

(Remarks on code availability)

Reviewer #5

(Remarks to the Author)
I co-reviewed this manuscript with one of the reviewers who provided the listed reports. This is part of the Nature Communications initiative to facilitate training in peer review and to provide appropriate recognition for Early Career Researchers who co-review manuscripts.

(Remarks on code availability)

Open Access This Peer Review File is licensed under a Creative Commons Attribution 4.0 International License, which permits use, sharing, adaptation, distribution and reproduction in any medium or format, as long as you give appropriate credit to the original author(s) and the source, provide a link to the Creative Commons license, and indicate if changes were made.
In cases where reviewers are anonymous, credit should be given to 'Anonymous Referee' and the source.
The images or other third party material in this Peer Review File are included in the article's Creative Commons license, unless indicated otherwise in a credit line to the material. If material is not included in the article's Creative Commons license and your intended use is not permitted by statutory regulation or exceeds the permitted use, you will need to obtain permission directly from the copyright holder.
To view a copy of this license, visit https://creativecommons.org/licenses/by/4.0/

BLUE: reviewer comments
BLACK: response to reviewer comments
Purple: New text in the manuscript

Reviewer #1
The manuscript entitled "Semi-supervised Omics Factor Analysis (SOFA) disentangles known and latent sources of variation in multi-omics data" proposes very interesting problem in multi-omics latent factor analysis. Given the increasing availability of multi-omics datasets out of several disease studies, it is extremely important to develop new statistical and analytical methods exploring the hidden structure and variation in such datasets to uncover and understand the novel biology. Most of the previous studies carried out unsupervised studies, and a few supervised studies, to identify the latent structure. SOFA proposes a semi-supervised method that utilizes known factors as guiding variables (e.g. disease subtype, gender, age, batch effects etc) to identify unknown hidden structure (latent factors) in the data. The main idea of the method proposed here is to regress out the known drivers of technical or known biological sources of variation from the latent space so that remaining hidden factors can be better identified. The method builds upon previously proposed Multi-Omics Factor Analysis (MOFA) and generalized regression with known covariates which the authors call as guiding variables.

Overall, the method proposed in the manuscript is very interesting, very well written, and is likely to contribute to the literature. The code is well written and well documented, and likely to be useful for users with a strong statistical background with expertise in Python. However, I feel that a few more clarification and elaboration is needed before publication.

We thank the reviewer for their time and effort they spent on our manuscript, and for their positive feedback.

Below are some of my comments:

The guiding variables (covariates) are modelled as a function of guided variables factors) in the model. It is not clear if the authors consider one guiding variable and one guided vector per function e.g. g1, g2 etc (Fig1b and methods section), or if more than one guided variable can be modelled within those functions. It is common to have fixed and only a few guiding variables in most cases. However, guided factors can be one or more depending on the data. I recommend authors to clarify that in the manuscript.

We thank the reviewer for this helpful comment and the opportunity to clarify. We have revised the Methods section (lines 512ff) to make this clearer:
"While there is a one-to-one relationship between guiding variables Y^c and inverse link functions g_c, their arguments depend, via β_c—depending on the user's choice—, on multiple guided factors Z."

How is the optimum number of factors determined? The identification of the number of factors with justification should be provided/elaborated in the methods section.

We thank the reviewer for highlighting this tricky question. We have edited the text (lines 561ff) as follows:
“We recommend looking at the Akaike (AIC) and Bayesian Information Criteria (BIC), which aim to balance goodness-of-fit with model complexity.”
…
“However in practice, similar to other unsupervised methods, such as clustering (number of clusters), PCA (number of principal components), it is not straightforward to choose the optimal number of factors solely based on model selection metrics, and we recommend looking for utility and interpretability of factors in terms of domain knowledge.”

For simulated data that were generated by a factor model, we added a set of evaluations of these criteria with SOFA in **Supplementary Figure S6b**. However, real data tends to be generated in more complex ways, and we point out in the revised paper that this question is, in essence, a common model selection problem that recurs similarly across many unsupervised methods, e.g., also in clustering (how many clusters), principal component analysis (how many principal components). After more than 70 years of work on it, statisticians now generally recommend using multiple methods (e.g., BIC, AIC) and looking for utility and comparison to domain knowledge, rather than relying on a single, formalized approach. This takes into account the fact that for many real datasets, there is no universally “right” number of factors, and that the usefulness of the factor decomposition tends to be context-dependent or even subjective.

While incorporation of the known a few covariates in the SOFA method performs reasonably well in the pattern recognition, adjustment of too many covariates may shrink true hidden pattern of interest and the hidden signal may not be identifiable in the residual data. This could be the limitation of the approach.

We agree with the reviewer’s thoughtful comment, and added the following statement to the Discussion of the revised manuscript (lines 462ff):
“Inclusion of too many guiding variables can degrade the capacity of SOFA to discover latent factors. In particular, this can happen if one or several guiding variables are approximately (multi-)collinear with one or several latent factors of interest. In such cases, lack of power from finite data size can lead to masking of the latent factors(Holmes and Huber 2019). Analysts can explore such situations by repeating SOFA fits with and without certain guiding variables and then decide based on domain knowledge, or other practical considerations, which of the guiding or latent (multi-)collinear variables are the most relevant or suitable ones for modeling the data at hand.”

Authors have presented comparison of the proposed SOFA with existing MOFA methods using heart failure single cell data. I wonder how the comparisons would come out if they had compared the methods in other three examples too.

It is indeed important to compare MOFA in the other examples too, we thank the reviewer for highlighting this. We inferred the same number of factors using MOFA and compared the spanned subspaces to the subspaces spanned by an unguided SOFA model and to a guided SOFA model. **Supplementary Figure S6e** shows the RV coefficients between the two SOFA models and MOFA. We added the following text to the results (lines 387ff):
*"To assess whether SOFA's factor guidance identifies subspaces distinct from those of MOFA, we compared the RV coefficients of unguided and guided SOFA models to those of a MOFA model. As shown in **Fig. S6e** , the guided SOFA model exhibited lower RV coefficients than the unguided model, indicating reduced similarity to the MOFA subspaces."*

and to the methods (lines 733ff):
"The RV coefficient measures the similarity of two subspaces and can be seen as the multivariate analogue of the Pearson correlation coefficient. It is rotation-, permutation-, and sign-invariant and takes values between 0 and 1, where values closer to 1 indicate higher similarity(Robert and Escoufier 1976). It is calculated as:

$$RV(A,B) = \frac{tr(AA^T BB^T)}{\sqrt{tr[(AA^T)^2] tr[(BB^T)^2]}}$$

Where A and B correspond to the loadings of two SOFA models with different initializations."

(Remarks on code availability):

The code looks good. The code is written in Python and is usable for those who knows Python. It would have been more helpful for broader community if the code had been written in R. However, this is my personal opinion and has nothing to do with my review.

We thank the reviewer for the positive feedback on the code and for acknowledging its usability. We also love R! It is widely used in the statistical bioinformatics community, and we agree that integration with Bioconductor and interoperability with its data structures would facilitate upstream operations such as data import, wrangling and 'normalisation', as well as downstreams tasks such as visualization and biostatistical analysis. The reason that the current implementation is in Python has been to take advantage of its packages pytorch and Pyro for probabilistic modeling. A current trend in data science is towards integration of Python and R (e.g.: reticulate, quarto, Positron), and we aim to take advantage of this by providing R bindings.

Reviewer #2

The manuscript introduces Semi-supervised Omics Factor Analysis (SOFA), a novel computational method for analyzing multi-omic datasets by integrating known covariates into a factor analysis framework. SOFA partitions latent factors into guided components (linked to specified variables) and unguided components (capturing novel variation), aiming to disentangle known and unexpected patterns in biological data. The method is demonstrated across diverse datasets, including cancer (TCGA, DepMap), single-cell brain development, and heart failure, showcasing its ability to uncover biologically relevant insights. Strengths include its innovative semi-supervised approach, robust Bayesian implementation, and broad applicability. However, several areas require enhancement to strengthen its contribution prior to publication.

We thank the reviewer for their time and effort they spent on our manuscript. We also thank them for their positive feedback and for their constructive comments on improving the manuscript.

Comments:
(1) The manuscript compares SOFA to Multi-Omics Factor Analysis (MOFA) in the heart failure analysis (lines 314-359) and briefly references methods like Group Factor Analysis (GFA), Principal Component Analysis (PCA), Partial Least Squares (PLS), SPEAR, and DIABLO (lines 57-85). However, it lacks a comprehensive comparison with other semi-supervised or supervised multi-omics tools, such as iCluster or MuVI. While quantitative metrics (e.g., variance explained, lines 339-341, Figure S5d) are provided, a detailed discussion of how SOFA's guided factor strategy outperforms or complements these methods would better articulate its unique contribution. For instance, how does SOFA address specific limitations of MOFA's unsupervised nature or DIABLO's emphasis on known covariates?

We now have included MuVI, SPEAR and a SOFA model only guided by heart failure labels in our comparison (**Figure S5d**). We added the following text to the result section (lines 350ff):

"To see whether the differences between SOFA and MOFA in explaining sex-related variance were due to differences in regularization, we compared them with SOFA models guided only by HF. We found that these simpler SOFA models failed to capture factors with weak sex signals and explained less HF-related variance than the fully guided SOFA models in three of the five studies.

Next, we compared the fully guided SOFA model with a fully unsupervised MuVI model, which, unlike MOFA, uses a horseshoe prior as its regularization strategy. We found no factor from the MuVI model associated with sex across studies.

These comparisons suggest that the improved capture of low-signal features is driven by SOFA's guidance, rather than the differences in regularization between models. To compare the guidance strategy of SOFA to the ones used by alternative models that are able to introduce prior knowledge to factor reconstruction, we fitted two additional models using SPEAR and MuVI. SPEAR and MuVI models used sex labels and genes linked to the Y chromosome, respectively, to guide factor reconstruction. When comparing the guided-SOFA models to the SPEAR models across all datasets, we found that SOFA consistently recovered a greater fraction of variance associated with sex and age (one-sided t-test,

adjusted p-value < 0.05). Additionally, SOFA recovered a greater fraction of variance associated with HF in 2 out of 5 studies, performing worse in only one. Compared to the sex-guided MuVI model, SOFA recovered greater variance fractions associated with sex in three out of five studies (one-sided t-test, adjusted p-value < 0.05), with the two remaining ones performing similarly. SOFA, in addition, recovered a greater fraction of variance associated with HF in 3 out of 5 studies, performing worse in only one."

Furthermore, we added a brief discussion to the manuscript of how SOFA addresses specific limitations of unsupervised MOFA and DIABLO's emphasis on known covariates (lines 424ff):
"MOFA, due to its unsupervised nature, concentrates on high variance signals in the data. This can lead to low variance signals not being picked up by MOFA. SOFA's guided approach can help to extract such signals if they are associated with a known covariate. SPEAR can include guiding variables, but the guiding variables potentially influence the whole latent space and are not assigned to specific factors, effectively separating known variance from unknown. MuVI uses a similar regularization strategy as SOFA (horseshoe priors), but in contrast to SOFA, but it answers a different question: it integrates prior feature groupings through structured sparsity priors, and thus aligns latent factors with predefined biological sets to enhance interpretability, but it does not have the more explicit covariate supervision that SOFA provides."

(2) SOFA relies on user-specified guiding variables (e.g., cancer types, cell labels) to structure its factors (lines 103-116), which may limit its utility when covariates are unclear or unavailable. The authors should acknowledge this constraint and consider suggesting strategies to identify potential guiding variables—such as unsupervised clustering or data-driven covariate selection—to enhance the method's flexibility.

We thank the reviewer for this important suggestion to improve the manuscript. We added the following text to the Discussion (lines 469ff):
"SOFA can also be applied if no guiding variables are apparent, in which case it effectively reduces to an unsupervised factor model, similar to MOFA (up to regularization, symmetries and numerical implementation details) In a subsequent step, users may study the discovered latent factors and decide to identify—by correlation—one or several of them with previously overlooked, explicit variables, and then make a renewed call to SOFA, now with these explicit variables as guiding variables. Similarly, users may first use other unsupervised methods to discover important guiding variables, and then use SOFA to do a factor analysis on the remaining variation."

(3) The use of stochastic variational inference supports scalability (lines 117-123), but the manuscript does not evaluate SOFA's performance with noisy or incomplete multi-omic data (e.g., missing modalities or technical artifacts). Including a robustness analysis under simulated noise conditions, building on the existing simulation framework (lines 466-478, Figure S1a), or discussing the impact of data quality on factor accuracy would bolster its practical credibility.

We thank the reviewer for this insightful suggestion. We have extended our simulation experiments to evaluate SOFA's robustness to incomplete data by introducing missing values into the observed data matrix. These new experiments simulate varying levels of

missingness and assess SOFA’s ability to recover the ground truth in comparison to a baseline (mean) and MOFA.

The results, presented in **Supplementary Figure 6a**, demonstrate that across a range of missingness levels, SOFA performs on par with the baseline and MOFA in terms of reconstruction error (RMSE). This supports its technical robustness in multi-omic settings where incomplete modalities or technical dropouts are common. However, we note that missing data can lead to difficult modeling issues, for instance if the missingness is correlated (e.g., a whole view is missing for a subset of samples, not just individual numbers), or if it is not at random (e.g., some assays may be missing, or present, only for patients with a particular version of the disease, due to clinical necessities).

We updated the Methods and Results sections accordingly to describe these additional simulations (lines 601ff):
*“Like MOFA, the SOFA model can be fit if subsets of the data are missing (e.g., if some views were only obtained for a subset of samples). The recovery of latent factors is fairly robust to missingness at random (**Fig. S6a**). However, we note that in practice, data missingness is often correlated and informative, and analysts need to mitigate missingness carefully. “*

(4) SOFA outperforms MOFA in detecting low-variance signals, such as sex (lines 339-341, Figure S5d), attributed to its guided framework. However, the manuscript does not fully explore why MOFA underperforms beyond its unsupervised design. A deeper analysis of how SOFA’s hierarchical Bayesian model or sparsity-inducing priors (lines 117-123) contribute to this advantage would clarify its technical superiority.

We thank the reviewer for pointing this out. We expanded our comparison of SOFA and MOFA to a SOFA model that was only guided by heart failure labels (**Supplementary Figure S5d**). We added the following text to the results section (lines 350ff):
“To see whether the differences between SOFA and MOFA in explaining sex-related variance were due to differences in regularization, we compared them with SOFA models guided only by HF. We found that these simpler SOFA models failed to capture factors with weak sex signals and explained less HF-related variance than the fully guided SOFA models in three of the five studies.”

(5) The adoption of a hierarchical horseshoe prior and variational inference is central to SOFA (lines 117-123, Figure 1a), yet the manuscript justifies these choices primarily for scalability and sparsity without comparing them to alternatives (e.g., Gaussian priors, Markov Chain Monte Carlo [MCMC]). Providing a theoretical or empirical rationale—such as their specific role in enhancing guided versus unguided factor separation—would reinforce the method’s design.

We thank the reviewer for this thoughtful suggestion. The adoption of the hierarchical horseshoe prior and variational inference in SOFA was motivated by practical and methodological considerations.
We opted for the hierarchical horseshoe prior as it induces sparsity, which in particular in high-dimensional settings is often desired by domain scientists, and aligns with biology-based concepts of modularity, where the whole system, e.g., a cell or a tissue, has

subsystems (“pathways”, organelles) that to some approximation operate autonomously and interact with each other through a smaller number of channels. We acknowledge, however, that this is a modeling choice, and other approaches may also lead to useful results.
We chose variational inference to ensure scalability to large datasets and complex models, whereas MCMC would—to the best of our knowledge—be computationally prohibitive for our intended use cases.

We have now added a discussion in the Methods section elaborating on the rationale behind these modeling choices (lines 541ff and 556ff).
“We use a hierarchical horseshoe prior[(Carvalho et al. 2009)](#) *to induce sparsity in the loading weights, which tends to facilitate model interpretation in high dimensional settings. However, we note that opting for the horseshoe was purely a choice of convenience, and other sparsity inducing approaches may also lead to useful results. “*
…
“Although variational approximations can introduce bias, they allow efficient posterior inference on large datasets where fully Bayesian methods like MCMC are computationally infeasible.”

(6) While SOFA’s implementation in Pyro is highlighted (lines 400-403), details on computational demands—such as runtime, memory usage, or scalability limits—are omitted. Including this information would enable users to assess its feasibility for large datasets, particularly in single-cell contexts with extensive sample sizes (e.g., lines 265-302, Figure 4).

We thank the reviewer for pointing out the importance of assessing computational demands. In response, we conducted additional experiments to evaluate SOFA’s resource (runtime, memory) usage.

These new experiments, presented in **Supplementary Figure S6c**, systematically measure SOFA’s performance across varying data set sizes and number of guiding variables. The results show that SOFA (a) scales linearly with dataset size and (b) uses resources available to a typical bioinformatics lab for typical relevant datasets.
We have updated the Methods and Results sections (lines 610ff) to describe these scalability experiments and to guide users on expected resource requirements.
“We evaluated SOFA’s scalability using simulated datasets with three views, two guiding variables and 10 factors while increasing the number of samples. Fig. S6c shows that datasets with up to 150k samples (or cells) can be processed in ~30 minutes, requiring ~4 GB of memory on standard GPUs.”

(7) The technical definitions of guided and unguided factors are well-explained (lines 433-438), but their biological implications could be more accessible. For example, providing intuitive interpretations of findings like Factor 10 in TCGA (lines 167-189, Figures 2c-e)—such as “high values reflect increased cell proliferation, low values indicate immune activity”—would broaden the manuscript’s appeal to non-specialists.
We thank the reviewer for this thoughtful suggestion. We agree that providing more intuitive, biologically grounded interpretations of the factors make the manuscript more accessible to a broader audience. In response, we have revised the relevant Results section (lines 182ff):

“High values of Factor 10 were associated with increased expression of proliferation-related genes and oncogenic pathways, while low values were enriched for immune-related signatures, suggesting an antagonism between tumor proliferation and immune activity. “

Reviewer #2 (Remarks on code availability):

The code is provided as an open-source Python package on GitHub and PyPI, with documentation and examples, facilitating reproducibility and adoption. Enhancing the documentation with tutorials that interpret typical outputs (e.g., factor distributions in Figure 2a or loading significance in Figure 3e) would further assist users in applying and understanding SOFA effectively.

We thank the reviewer for the positive feedback and the constructive suggestion. We agree that enhancing the documentation with tutorials that guide users in interpreting SOFA’s outputs supports effective application and understanding of the method.

In response, we have added similar plots such as in Figures 2a and 3e to the DepMap data analysis tutorial. Furthermore we added a new tutorial to the documentation that walks users through the interpretation of SOFA outputs using the TCGA dataset. This includes guidance on visualizing and interpreting factor distributions, examining covariate associations, survival analysis and assessing the relevance of factor loadings. We believe this addition will significantly improve the accessibility of SOFA for a wider range of users.

The updated documentation is available on the GitHub repository (https://github.com/tcapraz/SOFA) and linked in the manuscript.

Reviewer #3

The authors propose SOFA, a semi-supervised probabilistic factor model designed to integrate multi-modal omics datasets, combining unsupervised latent structure discovery with guided supervision based on known clinical or biological covariates. The authors then proposed multiple experiments to show the effectiveness of the proposed SOFA on multiple applications and scenarios. SOFA is benchmarked against MOFA and demonstrates improved performance, particularly in scenarios involving small sample sizes and weak biological signals. This paper is clearly written and easy to understand. All figures are well demonstrated and with suitable annotations.

We thank the reviewer for their time and positive feedback.

Comments:

1. SOFA only considers first-order features, ignoring potential two/higher order features. The authors implicitly assume that different guided features are independent, thus the guidance of factors to covariates is modelled via simple linear regression and there is no explicit modeling of nonlinearities, higher-order interactions. This assumption may not always hold in complex biological systems, which suggests that the unguided factors recalled by SOFA might be higher-order combinations of guided factors. The authors propose a simple experiment comparing the correlation between unguided factors with two/higher order combinations of guided factors.

We thank the reviewer for raising this insightful and salient point. We consider this a feature, not a bug. Finding latent (unguided) factors that are higher-order combinations of guided factors, or residuals from fitting a linear relationship to one that is truly non-linear (e.g., because of saturation) is a not infrequent outcome of SOFA. This is informative and potentially valuable to the user. If, after inspection, a user decides that she would prefer to explicitly model such higher-order combinations or non-linear functional forms, she can do so by extending or updating the guided variables, e.g., by adding the product of two existing guiding variables as another guiding variable. We updated the text in the methods to clarify this (lines 528ff):

"In Equation (2), the guided features $\beta_1, \ldots, \beta_C$ combine linearly into the arguments of the inverse link functions $g_1, \ldots, g_C$. If the link function g_c is the identity, the overall relationship between the mean parameter for the c-th guiding variable ν_i^c and the latent guided factors is linear. Based on the chosen likelihood, non-linearities can be modeled with the link functions. Conversely, users may choose to transform some of the guiding variables Y_c, e.g., by multiplying two guiding variables to model an interaction term between them."

Furthermore, we now discuss the biological interpretation of modeling interactions explicitly (Discussion lines 449ff) :

"If there is reason to assume that interactions between two or more guiding variables play a role, then users can simply augment the set of guiding variables and define additional ones, e.g., by multiplying two guiding variables to model an interaction between the two variables, or indeed using any other non-linear function of two or more variables. Examples may include gene drug-interactions such as TP53 mutation and sensitivity to Nutlin, or BRAF V600E mutation and sensitivity to vemurafenib.

On the other hand, if such interactions are not on the analysts' mind upfront, they may fit a SOFA model with a given set of guiding variables and then find an unsupervised factor that matches a non-linear function of one or several guiding variables. In such cases, they may take this finding forward directly, or define a new guiding variable that expresses this interaction, and refit SOFA to find further, perhaps yet more subtle latent factors."

2. No orthogonality constraint is imposed between guided and unguided factors, unguided factors may inadvertently absorb residual components of guided variables, potentially confounding biological interpretation.

We thank the reviewer for their feedback. We acknowledge that orthogonality is a popular choice to enforce non-redundancy, but we also note that there is no reason that in real systems, important underlying mechanisms are orthogonal. In SOFA, we encourage non-redundancy between the factors by asserting a diagonal covariance matrix for the multivariate Gaussian prior and by limiting the number of factors. Thus, factors are encouraged to explain different sources of variation, and in practice are often close to orthogonal. **Supplementary Figure S7a-c** shows the angles between factors for the three example datasets.

As for the possibility that unguided factors may inadvertently absorb residual components of guided variables, this is closely related to Point 1 above, and we have addressed this there.

3. The authors utilized a hierarchical horseshoe prior to shrink the loading weights on the view, which naturally makes me wonder about the performance of utilizing L1-normalization (LASSO) instead of horseshoe prior. The authors should perform further experiments validating this initialization method.

We thank the reviewer for their feedback. SOFA is formulated as a Bayesian hierarchical model and uses Bayesian Stochastic Variational Inference to fit its parameters. The LASSO corresponds to a Laplace prior and is typically used for MAP estimation rather than full Bayesian inference. In contrast, the horseshoe prior is a fully Bayesian global–local shrinkage prior that allows for posterior uncertainty quantification and better adaptive shrinkage of sparse signals. Since our model is fully Bayesian, the horseshoe prior is more appropriate for our hierarchical model. Implementing a LASSO equivalent would amount to changing the inference framework (to penalized likelihood or MAP), which is beyond the scope of this manuscript. However, we acknowledge the choice of the horseshoe prior is a choice of convenience; we consider it plausible that equally useful results could be obtained using the LASSO.

4. The model selection strategy is not described enough, as the selection of the number of factors is indeed the most important hyperparameter in SOFA. This manuscript does not provide us a reproducible or standardized procedure for model selection, nor how these parameters used in these analyses are chosen, which may greatly limit the usage of proposed SOFA.

We thank the reviewer for raising this important point. Selecting the number of factors is indeed crucial for using SOFA effectively. We have revised the text (lines 573ff) to

recommend evaluating the Akaike (AIC) and Bayesian (BIC) Information Criteria, which balance model fit and complexity.

For simulated factor-model data, we added corresponding AIC and BIC evaluations in Supplementary Figure S6b. For real data, where generative processes are more complex, we note that this is a common model selection challenge, similar to choosing the number of clusters or principal components in other unsupervised methods. In our analyses, we fitted several SOFA models with varying factor numbers and complemented AIC/BIC with manual inspection of biological interpretability. We now recommend combining such domain knowledge with information criteria for practical use (lines 561ff).
"We recommend looking at the Akaike (AIC) and Bayesian Information Criteria (BIC), which aim to balance goodness-of-fit with model complexity."
…
"However in practice, similar to other unsupervised methods, such as clustering (number of clusters), PCA (number of principal components), it is not straightforward to choose the optimal number of factors solely based on model selection metrics, and we recommend looking for utility and interpretability of factors in terms of domain knowledge."

5. Not all unguided factors identified by SOFA are clearly explained or linked to known biological processes. For example, in the TCGA analysis, Factor 9 shows a pattern very similar to Factor 10, yet it is not discussed in the paper. Overall, the biological relevance of many unguided factors remains unclear. Are these factors capturing novel biological signals, or are they just modeling noise? It would be important for the authors to clarify how they distinguish meaningful factors from noise.

We thank the reviewer for highlighting this, it is indeed important to distinguish between factors modeling technical noise or capturing biological signals. The unguided factors we did not discuss in our examples could indeed be relevant, however for the scope of this manuscript we decided to focus on factors that explain a comparatively high fraction of variance across all modalities. In practice this is usually a good way of prioritizing factors for deeper inspection. We added the following to the discussion section to clarify our analysis strategy (lines 439ff) :
"SOFA's unguided factors are comparable to factors from other factor analysis models such as MOFA and is thus a technique for exploratory data analysis. To discern unguided factors that pick up technical variation from biologically relevant factors, users need to inspect their loadings and correlate them with additional knowledge (e.g., disease subgroups). It may also be instructive to see whether they might reflect interactions between other factors or residuals from non-linear relationships."

6. The scalability of SOFA on large-scale datasets was not reported in this paper.
We thank the reviewer for raising this important issue. We performed simulations that evaluate the runtime and memory usage of SOFA and show their results in **Supplementary Figure S6c** and added the following text to the manuscript (lines 610ff):
"We evaluated SOFA's scalability using simulated datasets with three views, two guiding variables and 10 factors while increasing the number of samples. ***Fig. S6c*** *shows that datasets with up to 150k samples (or cells) can be processed in ~30 minutes, requiring ~4 GB of memory on standard GPUs."*

Reviewer #3 (Remarks on code availability):

The code and documentation seem straightforward to follow.

Reviewer #4 (Remarks to the Author):

I co-reviewed this manuscript with one of the reviewers who provided the listed reports. This is part of the Nature Communications initiative to facilitate training in peer review and to provide appropriate recognition for Early Career Researchers who co-review manuscripts.

In this study, Capraz et al. introduce Semi-supervised Omics Factor Analysis (SOFA), a hierarchical Bayesian factor model designed to disentangle known covariates from novel axes of variation in multi-omic data. This work represents a substantial improvement over existing unsupervised approaches like MOFA by explicitly modeling sample metadata to distinguish biologically meaningful (surprising) factors from expected covariate-driven factors.

Major feedback:

- The manuscript clearly demonstrates SOFA's advantages over MOFA, addressing well-known challenges such as interpretation of latent factors. However, a more direct comparison with supervised matrix decomposition methods (DIABLO, SPEAR) would strengthen the argument for SOFA's unique advantages. Providing empirical evidence or a clear discussion contrasting these supervised approaches would further clarify the distinct contributions of SOFA.

We thank the reviewer for pointing out that the distinction of SOFA to supervised methods DIABLO and SPEAR is not clear. We added the following to the discussion (lines 406ff):
"While fully supervised matrix decomposition methods such as DIABLO and SPEAR infer latent factors that are predictive of a response (or guiding) variable of interest, SOFA explicitly partitions the latent factors into supervised (or guided) factors and unsupervised (unguided) factors."

- The manuscript lacks detailed discussion about critical methodological decisions. Clarifying the choice and determination process for the number of latent factors, as well as elaborating on practical computational considerations (e.g., runtime, memory requirements), would significantly enhance reproducibility and practicality.

We thank the reviewer for raising this important issue. We have edited the text (lines 562ff) to advise users to look at the Akaike (AIC) and Bayesian Information Criteria (BIC), which balance goodness-of-fit with model complexity. For simulated data that were generated by a factor model, we added a set of evaluations of these criteria with SOFA in **Supplementary Figure S6b**. However, we note that real biological data tend to be generated in more complex ways, and in practice it is beneficial to combine information criteria with domain knowledge and manual factor inspection.
"We recommend looking at the Akaike (AIC) and Bayesian Information Criteria (BIC), which aim to balance goodness-of-fit with model complexity."
…
"However in practice, similar to other unsupervised methods, such as clustering (number of clusters), PCA (number of principal components), it is not straightforward to choose the optimal number of factors solely based on model selection metrics, and we recommend looking for utility and interpretability of factors in terms of domain knowledge."

Regarding resource requirements, we have now conducted additional experiments to evaluate SOFA's resource (runtime, memory) usage and show their results in **supplementary Figure S6c.** Additionally we added the following text to the manuscript (lines 610ff):

"We evaluated SOFA's scalability using simulated datasets with three views, two guiding variables and 10 factors while increasing the number of samples. ***Fig. S6c*** *shows that datasets with up to 150k samples (or cells) can be processed in ~30 minutes, requiring ~4 GB of memory on standard GPUs."*

- The authors briefly mention scalability ensured through stochastic variational inference but omit benchmarks or performance metrics. Including these details would aid readers in assessing SOFA's applicability to large and high-resolution datasets.

We thank the reviewer for their feedback. As mentioned in our response in the previous comment, we performed simulation experiments with up 150 000 samples and found that it took approximately 30 minutes to fit SOFA, highlighting its applicability to interactive analyses of large and high-resolution data sets.

- The paper would benefit from explicitly addressing the robustness of variational inference, including information on multiple initializations and convergence diagnostics. This detail is crucial to instill confidence in the reproducibility of the inferred factors.

We thank the reviewer for pointing this out, reproducibility of the inferred factors is indeed crucial to have confidence in SOFA's results. We added another simulation where we repeatedly fit a SOFA model on the same simulated data with different initializations and compare the subspaces spanned by the factors using the RV coefficient. An RV coefficient equal to 1 means identical subspaces. We included a plot of the results in **Supplementary Figure S6d**. We added the following to the results (lines 605ff):

"To check whether multiple initializations of SOFA models find essentially the same subspace, we fitted 10 SOFA models with the TCGA, DepMap, single-cell multiome and a simulated data set with 3 views and 2 guiding variables and calculated the RV coefficient to one of the initializations. ***Fig. S6d*** *shows that RV coefficients were close to 1 for all four data sets and indicates that SOFA yields consistent subspaces between different initializations."*

and to the methods (lines 733ff):

"The RV coefficient measures the similarity of two subspaces and can be seen as the multivariate analogue of the Pearson correlation coefficient. It is rotation-, permutation-, and sign-invariant and takes values between 0 and 1, where values closer to 1 indicate higher similarity[95]. It is calculated as:

$$RV(A,B) = \frac{tr(AA^T BB^T)}{\sqrt{tr[(AA^T)^2]tr[(BB^T)^2]}}$$

Where A and B correspond to the loadings of two SOFA models with different initializations."

- A clearer explanation of the statistical assumptions (e.g., Gaussian likelihood assumptions and linear relationships between guided factors and covariates) would improve

methodological transparency. Clarifying if SOFA can accommodate non-Gaussian modalities or nonlinear relationships would enhance its perceived flexibility.

We thank the reviewer for pointing out that we did not clarify the statistical assumptions behind SOFA. We added the following to the first results section (lines 108ff):
"SOFA models each omics view with a Gaussian likelihood and therefore expects omics data appropriately normalized and transformed. For the guiding variables users can choose between Bernoulli (binary), Gaussian (continuous) and Categorical (categorical) likelihoods."

- Critical methodological details mentioned in the online documentation, such as the adjustment of learning rates and the scaling factor for supervision strength, should also be explicitly described in the methods section of the manuscript.

We thank the reviewer for their feedback. We added the following text to the methods section (lines 574ff):
"SOFA allows users to specify the learning rate for model fitting. We recommend initializing the learning rate at 0.05 for the first 3,000 epochs and gradually decreasing it to 0.001 over the final 3,000 epochs. In practice, optimal learning rates and epoch numbers depend on the dataset, and convergence can be monitored using the ELBO. To guide users, we implemented an early stopping criterion, which stops training when improvement of the ELBO loss is lower than a user chosen percentage.
To control the supervision strength of guiding variables, SOFA enables users to set a scaling factor for each variable. This factor adjusts the corresponding likelihood term, increasing emphasis on explaining the guiding variable. Higher scaling factors enforce stronger supervision, while a value of zero removes supervision entirely. We empirically found that a default value of 1% of the total number of features in the omics views leads to a good tradeoff between supervision and explained variance. This scaling factor was also used for all our analysis results."

Minor feedback:

- The manuscript exhibits inconsistencies in spelling (US vs. UK English); uniformity would enhance readability.

We thank the reviewer for pointing out the inconsistencies, we changed all relevant words to US English.

- The rationale behind choosing visualization methods (tSNE vs. UMAP) for figures 3 and 4 is not clearly stated. Clarifying these choices would improve the manuscript's coherence.

We thank the reviewer for highlighting this. We only used tSNE and UMAP for visualization purposes and did not use the coordinates for further analyses. In principle both algorithms would be equally applicable. We added the following text to the methods (lines 713ff):
"We chose to visualize the latent space of the TCGA and DepMap data sets using tSNE and UMAP for the single-cell multiome data set. This choice was made for visualization clarity, as UMAP produced more interpretable plots for data sets with larger numbers of data points."

- SOFA's specification of Python=3.8 raises concerns due to its end-of-life status. Clarifying compatibility with current Python versions would support broader adoption.

We thank the reviewer for bringing this to our attention. The specification Python=3.8 in our installation documentation was an example. The actual requirement is Python <3.11.6, >=3.8.1. We have clarified this in the documentation of the package.

- While the manuscript effectively validates guided and unguided factors through biological interpretation, additional external or prospective validation would strengthen claims of biological novelty. Discussing the potential or strategy for validating unguided factors using independent datasets could address this.

We thank the reviewer for their feedback. Validation of Factors of interest using independent datasets is indeed important, especially in translational research. We added the following to the discussion (lines 444ff) including a reference to prior work from our lab using a validation cohort for a discovered MOFA factor (Lu, J., Cannizzaro, E., Meier-Abt, F. *et al.* Multi-omics reveals clinically relevant proliferative drive associated with mTOR-MYC-OXPHOS activity in chronic lymphocytic leukemia. *Nat Cancer* **2**, 853–864 (2021). https://doi.org/10.1038/s43018-021-00216-6):
"In practice, factors should be validated in independent datasets, or by mechanistic study, to instill confidence in their generalizability. Penalized multivariate regression on the validation can be used as outlined in (Lu et al. 2021) to predict the factors of interest."

- The authors should acknowledge limitations such as potential complexity in selecting guiding variables and interpreting guided factors, as well as explicitly discuss the implications of the linear modeling assumption for covariates.

It is indeed important to discuss how guiding variables should be chosen. We added the following text to the manuscript to guide the users in selecting appropriate guiding variables (lines 469ff):
"SOFA can also be applied if no guiding variables are apparent, in which case it effectively reduces to an unsupervised factor model, similar—up to regularization and symmetries—to MOFA. In a subsequent step, users may study the discovered latent factors and decide to identify—by correlation—one or several of them with previously overlooked, explicit variables, and then make a renewed call to SOFA, now with these explicit variables as guiding variables. Similarly, users may first use other unsupervised methods to discover important guiding variables, and then use SOFA to do a factor analysis on the remaining variation."

Regarding the implications of the linear modeling assumption for covariates, upon further reflection and a comment by Reviewer 3, we have now added additional text to the methods (lines 528ff):
In Equation (2), the guided features $\beta_1, \ldots, \beta_C$ combine linearly into the arguments of the inverse link functions $g_1, \ldots, g_C$. If the link function g_c is the identity, the overall relationship between the mean parameter for the c-th guiding variable ν_i^c and the latent guided factors is linear. Based on the chosen likelihood, non-linearities can be modeled with the link functions. Conversely, users may choose to transform some of the guiding variables Y_c, e.g., by applying a logarithm or square root transformation.

And discuss the biological interpretation of modeling interactions explicitly (Discussion lines 449ff) :
"If there is reason to assume that interactions between two or more guiding variables play a role, then users can simply augment the set of guiding variables and define additional ones, e.g., by multiplying two guiding variables for interaction of two variables, or indeed any other non-linear function of two or more variables. Examples may include gene drug-interactions such as TP53 mutation and sensitivity to Nutlin, or BRAF V600E mutation and sensitivity to vemurafenib.

In summary, Capraz et al. present an innovative and valuable methodological contribution to multi-omics factor analysis. Addressing these additional methodological, comparative, and interpretational considerations will further strengthen the manuscript's clarity, impact, and utility to the community.

Reviewer #4 (Remarks on code availability):

The documentation of the code is sufficient. Some overall questions regarding code are also included in the comments to the authors.

We thank the reviewer for their time and effort they spent on our manuscript, and for their positive feedback.
